# Chinese Americans’ Views and Use of Family Health History: A Qualitative Study

**DOI:** 10.1371/journal.pone.0162706

**Published:** 2016-09-20

**Authors:** Lei-Shih Chen, Ming Li, Divya Talwar, Lei Xu, Mei Zhao

**Affiliations:** 1 Department of Health and Kinesiology, Texas A&M University, College Station, Texas, United States of America; 2 Department of Health Education and Promotion, East Carolina University, Greenville, North Carolina, United States of America; 3 Department of Public Health, University of North Florida, Jacksonville, Florida, United States of America; Harvard Medical School, UNITED STATES

## Abstract

**Objective:**

Family health history (FHH) plays a significant role in early disease detection and prevention. Although Asian Americans are the fastest growing U.S. immigrant group, no data exists regarding Chinese Americans’ (the largest Asian subgroup) views and use of FHH. This study examines this important issue.

**Methods:**

Forty-nine adults from southern U.S. Chinese American communities participated in this qualitative, semi-structured, in-depth interview study. Interviews were audio recorded, transcribed, and analyzed with a content analysis approach.

**Results:**

Although the majority of participants perceived the importance of collecting FHH, most lacked FHH knowledge and failed to collect FHH information. Barriers affecting FHH collection and discussion among family members included long-distance separation from family members, self-defined “healthy family,” and Chinese cultural beliefs. Lack of doctors’ inquiries, never/rarely visiting physicians, self-defined “healthy family,” perceived insignificance of discussing FHH with doctors, and Chinese cultural beliefs were the obstacles in communicating FHH with physicians.

**Conclusions:**

Chinese Americans had limited usage of their FHH and faced cultural, distance, knowledge-, and healthcare system-related barriers that influenced their FHH use. Developing FHH education programs for Chinese Americans is highly recommended.

## Introduction

Family health history (FHH) is a genomic tool that represents the interactions among genes, behavior, and environment. An individual’s FHH plays a significant role in early disease detection and prevention [1, 2]. In recent times, FHH has been proposed as an “identification marker” to classify an individual’s disease risk level. Based on that risk level, health care providers can provide personalized disease prevention recommendations. This may reduce disease risk by motivating individuals to engage in healthy behaviors and to undergo early screenings in order to detect disease. Thus, the role of FHH in health care delivery is becoming increasingly important [1, 3].

To increase awareness of FHH among Americans, leading health agencies, such as the National Institutes of Health [4], the Centers for Disease Control and Prevention [5], and the U.S. Surgeon General [6] have diligently promoted the implementation of FHH initiatives and activities. Examples include the development of the “My Family Health Portrait” (the FHH collection tool) [6], the public advocacy of FHH discussion with physicians, and the promotion of Thanksgiving as the National FHH Day for encouraging FHH discussion among family members [7]. Moreover, studies have reported that Blacks and Hispanics families often do not systematical collect FHH due to challenges in gathering FHH information from family members, a lack of formal record keeping, and a limited awareness of the roles FHH plays in disease prevention [8–11]. Consequently, researchers have implemented successful FHH education programs for Blacks and Hispanics [10, 12–14].

Asian Americans are the fastest growing racial/ethnic minority in the U.S., with the population having increased by 43% from 2000 to 2010 [15]. Among them, the largest subgroup is Chinese Americans, who are mainly immigrants [15, 16]. Thus, physicians will likely interact with an increasing number of Chinese American immigrant patients. Previous studies [17–21] have found that certain aspects of Chinese culture, such as collectivism, influence Chinese Americans’ beliefs about health and illness, communication in health care, and the utilization of Western health care. Thus, physicians should consider their cultural beliefs in FHH collection and communication to provide culturally appropriate FHH-based services. Unfortunately, there are no published empirical FHH studies targeting Chinese Americans. The strongest evidence is the absence of baseline data regarding Chinese Americans’ views, collection, and communication of FHH. To address this important issue in genomics/FHH research, this first study aims at exploring Chinese Americans’ views and their FHH use.

## Methods

### Study Design, Recruitment, and Sampling

Initially, we discussed this research idea with leaders from two major Chinese American community-based organizations to obtain their input. These two Chinese American community-based organizations were non-profit organizations in the southern U.S. with the same joint mission of serving local Chinese Americans in the area and enhancing Americans’ understanding of Chinese culture. All of their members were Chinese American. With these leaders’ support, the first author (L.S.C.) and three members of these organizations (L.X., P.W., and M.L.L) recruited the initial sample of Chinese American participants from their networks. The eligibility criteria for participant recruitment were as follows: (1) participants must be Chinese Americans who originally came from China, Taiwan, or Hong Kong; and (2) participants must be 18 years old or older. The first author also trained L.X., P.W., and M.L.L. (all of whom were obtaining master’s degrees) in qualitative data collection techniques. The initial sample was diverse in socioeconomic status (SES). To reach a larger sample, we used a snowball sampling technique [22] to recruit additional interviewees.

### Data Collection and Measures

With input from community leaders and members, an interview guide was developed in both Mandarin and English. The first author, L.X., P.W., and M.L.L. conducted in-depth, semi-structured, one-on-one interviews in the preferred language of each participant. The interviewers first introduced the study background and then asked participants the following questions: (1) Who should be included in an FHH pedigree? (2) How accurately could you provide FHH to your doctors? (3) How important is it for you to collect FHH? (4) What are the barriers for obtaining and/or discussing FHH with your family members? (5) What are the obstacles in discussing FHH with your doctors? Field notes were taken during the interview to help interpret data [22].

Demographic information was collected from participants (i.e., age, gender, educational level, annual household income, number of children, and health insurance coverage). We assessed their acculturation, which was defined as “the degree to which Asian- [Chinese-] Americans are identified with and integrated into the white majority culture,” [23] (p. 198) by examining participants’ English language proficiency and residency in the U.S. based on the tool developed by Wang and colleagues [24]. Participants were designated as highly acculturated (1) if they had resided in the U.S. for ≥ 10 years and (2) if they self-reported their English proficiency as good/very good on a five-point scale in listening, speaking, reading, and writing skills. Interviews lasted for an average of 45 minutes. Data saturation was reached with 49 participants, with no more additional findings generated from interviews. We gave $10 gift cards and health education brochures as incentives.

### Data Analysis and Validation/Trustworthiness

Interviews were audio -recorded and transcribed verbatim by the three trained interviewers (L.X., P.W., and M.L.L.) in Mandarin (n = 48) and English (n = 1). The first author (also the interviewer) read and verified transcripts. Then, the first author trained two graduate students (one was an interviewer) who were fluent in Mandarin and English as coders for data analysis. As no literature exists on FHH use among Chinese Americans, we used the conventional content analysis approach [22, 25] to analyze data. With the assistance of NVivo 9, two coders (M.L. and L.X.) independently read the transcripts and identified codes emerging inductively from the interview data. Coders discussed the codes and collapsed those codes into subthemes, which responded to specific interview questions (i.e., themes). The consensus in these themes was discussed with the first author (L.S.C.) to reach an agreement. Extensive team discussion to reach an agreement in data analysis ensured the trustworthiness of the qualitative data [26]. Frequencies were counted by examining the number of times participants mentioned particular subthemes/themes. Moreover, during the data analysis, the research team collapsed two interview questions regarding “FHH collection” and “FHH discussion” from family members into one theme, given that participants could not differentiate between these concepts. To ensure the fidelity of the data analysis, we compared field notes to interview findings to identify if there are any discrepancies. These field notes also supplemented data interpretation [27].

### Ethics Statement

This study was approved by the Institutional Review Board at the University of North Florida. Written, informed consent was obtained from all participants before interviewing.

## Results

### Participant Characteristics

Table 1 shows the demographic information of the 49 participants. Their average age was 43 years, ranging from 18 to 75 years. Over half were females, and 59.2% had visited family doctors for healthcare. Most participants were married (79.6%), were college graduates or had higher education degrees (67.3%), and had health insurance (69.4%). Participants’ annual household income and religions preferences varied and the majority had low levels of U.S. acculturation (61.2%).

**Table 1:** Sample Characteristics of 49 Chinese American Interviewees.

Characteristics	n (%)
Age: mean ± SD (range)	43.0 ± 13.0 (18–75)
Gender	
Female	27 (55.1)
Male	22 (44.9)
Education	
High school diploma or less	14 (28.6)
Some college	2 (4.1)
College graduate or above	33 (67.3)
Marital Status	
Married	39 (79.6)
Single/divorced	10 (20.4)
Employment status	
Employed	40 (81.6)
Home maker	4 (8.2)
Student	2 (4.1)
Unemployed	2 (4.1)
Retired	1 (2.0)
Nativity	
Mainland China	30 (61.2)
Taiwan	13 (26.5)
Hong Kong	5 (10.2)
United States	1 (2.0)
Annual household income	
< $25,000	12 (24.5)
$25,000 to < $35,000	7 (14.3)
$35,000 to < $50,000	6 (12.2)
$50,000 to < $75,000	7 (14.3)
≥ $75,000	17 (34.7)
Religious affiliation	
No religious preference	23 (47.0)
Christian	15 (30.6)
Buddhist	8 (16.3)
Other	3 (6.1)
Health insurance coverage	
Yes	34 (69.4)
No	15 (31.6)
Having U.S. family doctors	
Yes	29 (59.2)
No	20 (40.8)
Level of acculturation	
Low	30 (61.2)
High	19 (38.8)

### Participants’ Knowledge of Their Own FHH Pedigrees

As a comprehensive FHH pedigree is comprised of medical information from at least three generations, we first asked participants about who should be included in their FHH pedigrees. Nearly all participants mentioned their parents, yet some also reported that they would not include parents in their FHH pedigrees. For example, one Chinese high school student told us that his brother had hypertension, but neither of his parents had this condition. He asserted that his parents’ medical history should not be included in the FHH pedigree since his brother’s hypertension was not inherited from either of his parents. Many participants ignored other first-degree relatives such as siblings and children. Though both grandparents and aunts/uncles are second-degree relatives, more participants acknowledged the need to collect FHH from their grandparents than their aunts/uncles. Few respondents recognized the importance of collecting FHH from first cousins (third-degree relatives).

Later, we gave an example of a comprehensive pedigree and informed participants that such a pedigree is comprised of medical information regarding the years of disease diagnoses, years of death, and causes of death from at least three generations. We then asked participants about the accuracy of the FHH that they could provide to physicians. Most respondents could not provide correct FHH, and several had no idea about their FHH.

### Importance of FHH Collection

Participants were asked about the importance of collecting FHH. The majority of them recognized the importance. Their supporting arguments included: disease prevention (e.g., “disease prevention…I was thinking about undergoing a physical examination to test the same disease my father has”); provision of useful information to the families (e.g., “FHH helped me to learn about the diseases that run in my family, which would be useful to my children, parents, and myself”); contribution to research because their FHH records might provide valuable information for current and future medical research (e.g., “helping FHH research”); and better disease diagnosis (e.g., “doctor could diagnose my disease based on the FHH”).

Nevertheless, several participants did not perceive the need for FHH collection. All of them reported that they had never thought about the idea of FHH collection in their lifetimes. One interviewee who obtained a Ph.D. in the U.S. reported:

“You don’t know what you can do after knowing your FHH. You don’t know what the purpose and use of FHH are, even if you have FHH information.” (Male, 32 years old, low acculturation, college graduate or higher, >75K, with health insurance)

Similarly, another participant, who was a pastor in a local Chinese church, stated that he did not foresee the value of collecting FHH because not all family-related diseases would pass on to the next generation. Further, a Chinese woman, whose two daughters were born in the United States, told us that she refused to think about her FHH because knowing about her FHH and discussing it with other people would upset her.

### Barriers for Obtaining/Discussing FHH from Family Members

We asked participants how often they collected or discussed FHH with their families. Some participants had never collected FHH. Among those who had collected FHH, however, most of them rarely discussed or collected FHH. Participants further identified the barriers preventing them from FHH collection below (Table 2).

**Table 2:** Perceived Barriers in FHH Collection and Communication with Family Members among Chinese American Participants.

Perceived barriers in FHH collection and communication with family members
Long distance and physical separation from family members
A self-defined “healthy family”
Chinese cultural beliefs
The perceived insignificance of discussing FHH with family members

Specifically, most participants believed the long distance and physical separation from their families hindered their FHH collection. Many of their relatives lived in their places of origin (i.e., China, Taiwan, or Hong Kong). Their communication channel was mainly via phone calls. Detailed discussion about FHH during these phone calls was not a priority for them. For instance, an interviewee who had lived in the U.S. for 30 years stated: *“Distance really separates me and my family in Taiwan*. *We often do not see each other*. *I seldom talk about diseases with them on phone calls” (Female*, *53 years old*, *high acculturation*, *college graduate or higher*, *>75K*, *with health insurance)*. The other leading factor affecting interviewees’ discussion and collection of FHH with family members was self-defining their own family as being “healthy.” Some respondents with diverse SES believed that their family members were healthy and that it was unnecessary to collect or discuss FHH information with their relatives. One participant explained: *“Everyone in my family*, *including the kids*, *are very healthy*. *It seems unnecessary to know FHH” (Female*, *50 years old*, *high acculturation*, *college graduate or higher*, *>75K*, *with health insurance)*. Similarly, another young participant reported that his parents never discussed FHH with him because they believed that everybody was healthy in his family.

Some participants (most with a low acculturation level) expressed that discussing diseases among families is considered a Chinese taboo. One interviewee disclosed that he would never ask his relatives about their disease histories. Asking about this information can be considered impolite in Chinese culture. Similarly, one participant thought that Chinese families avoid talking about FHH by saying: *“My parents thought it was unnecessary to tell something painful*, *like diseases*, *to kids…It is our Chinese culture” (Male*, *54 years old*, *high acculturation*, *college graduate or higher*, *>75K*, *no health insurance)*. Moreover, several interviewees mentioned that certain diseases, especially mental disorders, were culturally stigmatized. They believed Chinese Americans would not reveal such diseases to other family members. They would also keep their FHH of disease a secret in order to avoid being stigmatized by Chinese communities.

Lastly, not valuing the importance of FHH was identified by several interviewees as a barrier for discussing FHH with family members. These participants were unaware of the importance of FHH and did not know about the role that FHH could play in disease prevention. For example, one participant revealed: *“Knowing family health history*, *so what*? *What can you do*?*” (Male*, *32 years old*, *low acculturation*, *college graduate or higher*, *>75K*, *with health insurance)*

### Barriers to Communicating FHH with U.S. Physicians

Interviewees were asked whether they had discussed FHH with American doctors. Less than half had consulted with American doctors about FHH. Among these participants, most rarely communicated FHH to their doctors. We then asked participants to list the obstacles that affected their FHH communication with U.S. doctors. Their obstacles are described in Table 3.

**Table 3:** Perceived Barriers to Communicating FHH with U.S. Physicians among Chinese American Participants.

Perceived barriers to communicating FHH with U.S. physicians
Lack of inquiry from American doctors
Never or rarely visiting doctors in the U.S.
A self-defined “healthy family”
The perceived insignificance of discussing FHH with American doctors
Chinese cultural beliefs
Lack of FHH collection
A language barrier
Mistrust of U.S. doctors

In particular, the most common barrier recognized by participants was the lack of inquiry from American doctors. For example, one interviewee said: “*My [U*.*S*.*] doctor never asked me about it*. *I remember filling out a form about my own health status*, *and that’s it*. *I think when I go to see the [U*.*S*.*] doctor*, *he is only concerned about me*, *not my family” (Male*, *22 years old*, *high acculturation*, *high school*, *25K-35K*, *with health insurance)*. Similarly, several Chinese Americans reported that they have been asked to fill out FHH questionnaires, but their American doctors had never initiated a conversation regarding their FHH. One participant also had a misunderstanding about doctors’ role in the FHH communication. This participant believed that “not being asked about FHH from the U.S. doctors” is the doctors’ way of showing their “respect towards patients’ privacy.” In addition to the lack of inquiry from American doctors, some interviewees reported that they had rarely or never visited doctors in the U.S. Consequently, they hardly discussed FHH with their physicians. To these interviewees, visiting a U.S. doctor was a costly, inconvenient, and time-consuming process compared to doctoral visits in their birthplaces. One woman mentioned that it was too expensive to visit an American physician. She would rather visit physicians in Taiwan and hope that God would bless her by not getting sick in the U.S. Additionally, one participant who had a Health Maintenance Organization insurance plan stated that he had no family doctors, so he had never discussed FHH with doctors.

Several Chinese American participants assumed that it was unnecessary to discuss FHH with American doctors because they were healthy and their families had no severe FHH-related disorders. For example, one participant asserted that he was very healthy, so there was no need to discuss FHH with doctors. Three interviewees would discuss FHH with their American physicians only if they started aging or got sick from a serious ailment. For instance, one 18-year-old interviewee stated:*“I have no serious diseases at this moment*, *so there is no need to talk about FHH with my [U*.*S*.*] doctor*. *However*, *I will talk to doctors if I get serious diseases or become old” (Male*, *18 years old*, *high acculturation*, *high school*, *<25K*, *no health insurance)*. Further, several interviewees viewed FHH as unimportant and had no motivation to communicate with their American doctors regarding this issue. To them, it was neither an urgent nor a serious issue to talk about FHH with a physician. This viewpoint was illustrated by another participant: *“There is just no trigger for me to talk about my family diseases*. *I never thought it was important to mention it [FHH] to the [U*.*S*.*] doctor” (Female*, *32 years old*, *low acculturation*, *college graduate or higher*, *>75K*, *with health insurance)*. Participants also told us that the only time they needed to talk about FHH was during their first appointment with their American doctors. They believed it was unnecessary to discuss or mention FHH with doctors in subsequent visits.

Similar to the barrier of Chinese culture beliefs identified by some participants in FHH communication with family members, a few lowly acculturated interviewees recognized that sharing FHH with American doctors conflicted with Chinese cultural beliefs because “FHH-related diseases” were perceived as taboo. One participant elaborated: *“You know Chinese… we do not talk bad things of the family to others…sharing FHH with people outside the family is not a good thing” (Male*, *75 years old*, *low acculturation*, *college graduate or higher*, *<25K*, *with health insurance)*. A participant explained that she would discuss FHH with an American doctor only if deemed necessary, as talking about FHH conflicted with her Chinese cultural beliefs. As a result, she felt uncomfortable in disclosing and discussing her FHH information with her American doctors. Along with this Chinese cultural taboo, participants reported that they could not discuss FHH with their doctors because they did not have this information. As explained by a participant: *“I don’t really know my FHH*. *That is why I have never discussed it with my [U*.*S*.*] doctors” (Female*, *41 years old*, *low acculturation*, *college graduate or higher*, *<25K*, *no health insurance)*. Another Chinese American woman stated that she would be willing to share her FHH with American physicians, but she could not provide such information because she had never collected her FHH information.

Finally, a few participants identified language and mistrust of U.S. doctors as two more barriers in FHH communication with their American physicians. For example, though one participant had a college degree, he could not comprehend medical jargon. Another participant, who had lived in the United States for 17 years, reported that her U.S. doctor did not care about her health and stated: *“I think [American] doctors never have time to care about my health*. *They are not reliable” (Female*, *49 years old*, *high acculturation*, *college graduate or higher*, *>75K*, *no health insurance)*.

## Discussion

The majority of participants recognized that FHH provided useful information for themselves, their families, and research. This finding is consistent with national surveys [28, 29] that showed the majority of Americans believed in the importance of gathering FHH, but most failed to obtain their FHH. Even if participants obtained FHH, they often noted that the information was incomplete and/or inaccurate.

Lack of complete, accurate FHH from Chinese Americans may hinder clinicians from providing personalized screening and lifestyle recommendations [1, 3]. We explored several underlying reasons to explain the limited FHH collection among Chinese Americans. The leading factor was the lack of FHH awareness and knowledge. Nearly all respondents incorrectly answered the question about who should be included in an FHH pedigree. Further, some participants did not believe discussing or collecting FHH information with their family members was essential. Additionally, several interviewees assumed that their family members and themselves to be healthy and felt no need for FHH collection.

Moreover, physical separation and limited contact with family members were other barriers affecting participants’ FHH collection and discussion. This could be attributed to the fact that most Chinese Americans are immigrants [16]. This finding is in conjunction with another study on Latino immigrants: geographical distance is one of the barriers for them in obtaining and discussing FHH information with families in their country of origin [11]. Due to the lack of medical records in the U.S. and limited contact with distant family members from their birthplaces, collecting FHH is a challenging task for Chinese American immigrants. Immediate action is needed to promote FHH collection among this population. If no action is taken, future generations may encounter greater difficulties in obtaining FHH, due to the possibility of less frequent contact with family members residing outside of the U.S.

Notably, many interviewees identified physicians (i.e., lack of inquiries from physicians and mistrust in physicians) as well as the U.S. healthcare system (i.e., lack of Chinese translators and costly, inconvenient, and time-consuming processes of physician visits) as barriers affecting FHH discussion. The obstacle related to physicians is in line with the literature, which shows that U.S. physicians overall do not have sufficient competencies to communicate FHH with patients and deliver FHH-related services [30, 31]. Moreover, Asian Americans tend to mistrust U.S. healthcare providers [17]. The complexity of the U.S. health care system is a common challenge for disadvantaged groups [17, 32–34]. As physicians play an important role in patients’ FHH awareness, collection and discussion [35], it is crucial to train physicians in FHH-related practices and enhance their cultural competencies.

Several participants identified certain aspects of Chinese culture as an element that prevented them from collecting or discussing FHH with their families and U.S. physicians. As most participants who referred to this cultural stigma also had a low acculturation level, several factors might explain this phenomenon. First, in Chinese culture, which is known as a collectivist culture, family is often perceived as a single entity, which is considered to be more important than the individuals that comprise it [36]. Disclosing “family secrets (FHH-related diseases)” to other people can be perceived as disloyal. Second, some FHH-related diseases might be stigmatized in certain aspects of Chinese culture; therefore, Chinese Americans might be reluctant to disclose their FHH. Accordingly, culturally appropriate FHH education is needed to help Chinese Americans who face cultural barriers in family health history communication and/or collection with family members and doctors.

Interestingly, a self-defined “healthy family” is a common barrier identified by Chinese American participants in FHH collection/communication with family members and U.S. physicians. While this finding has not been reported in previous literature, it has an imperative educational implication. For example, some Chinese Americans may claim or believe that their families are healthy overall without thinking about specific diseases of each family member. Educational interventions may be developed to educate Chinese Americans regarding which diseases should be included in a comprehensive family history pedigree.

This study has several limitations. First, our participants were primarily first-generation immigrants. While most Chinese Americans are immigrants [16], examining the views of other generations is important. Second, participants (majority with a high SES) were recruited from two southern U.S communities. Our results might not represent all Chinese Americans’ views. Notably, as the high SES participants had already faced many barriers in FHH use, the low SES group might have encountered more obstacles. Our study contributes to the field as the first step to address this significant topic. Third, our results showed that Chinese cultural beliefs affected some participants’ family history communications with family members and health care providers. However, it is important to note that the views of individuals who identify as Chinese Americans might vary in their sociodemographic characteristics and cultural beliefs. Fourth, FHH collection and discussion seemed to be different behaviors, and we asked separate questions during the interview. Nevertheless, participants could not differentiate between these behaviors. Therefore, we combined these two questions in the data analysis. Lastly, we aimed to examine FHH views from Chinese Americans. Obtaining data from physicians, especially those dealing with Chinese American patients, may enrich our findings.

## Conclusion

This community-based study represents an initial window to explore FHH views and use among Chinese Americans. As Chinese Americans are under-represented in FHH research [37], our study contributes to the understanding of their views and use of FHH. Although most participants recognized the importance of FHH, their usage of FHH was limited. Barriers related to cultural beliefs, distance, knowledge, and the healthcare system affected FHH use. To provide better FHH-related healthcare services, efforts are needed to educate physicians to improve both their genomic and cultural competencies when collecting and discussing FHH with Chinese American patients. Moreover, the U.S. government is currently promoting “My Family Health Portrait” a Web-based tool (https://familyhistory.hhs.gov) among Americans to support the collection and usage of FHH. This Web-based tool may facilitate the FHH usage among Chinese Americans by: (1) educating them regarding the inclusion of diseases, relatives, and related information for a complete FHH pedigree; and (2) gathering FHH information electronically and sharing it with family members and physicians. As this Web-based tool currently only has English, Spanish, Portuguese, and Italian versions, development of a Chinese version is essential to promote health equity in genomics research. Lastly, as risk-stratified genetic and genomic tools that use FHH are developed and implemented, lack of comprehensive FHH from Chinese Americans may significantly affect disease prevention, detection, and treatment among this particular racial/ethnic group. The development of culturally and linguistically appropriate FHH education programs is a requirement for this fast-growing Asian American immigrant subgroup in the United States.

## Supporting Information

S1 File FHH Importance and Barriers. (ZIP)
