# Supplementary material for: Chinese Americans’ Views and Use of Family Health History: A Qualitative Study
Source: PLoS One. 2016 Sep 20;11(9):e0162706. doi: 10.1371/journal.pone.0162706 (PMC5029932; doi:10.1371/journal.pone.0162706)
Supplement: S1 File — (ZIP) [file pone.0162706.s001.zip › Data/Barriers to discuss with doctors/Chinese culture taboo and stigma.docx]

**Name:** Chinese culture taboo and stigma

**<Participant # 11. > - § 2 references coded [13.63% Coverage]**

**Reference 1 - 6.36% Coverage**

I:您从哪里得到的有关您的“家族病史”的信息？

P： 很小的时候从父母口中得知。因为我小时候吃一点（容易上火）的东西喉咙就疼。我妈妈跟我说我们的家族都这样不能吃太多。（I： 有看过医生吗？还是家里人这样说而已？） （I：有没有医生证明你不能吃这些东西？）医生也是知道的。医生能看出来的。医生不能说这个活，这是犯罪的。医生只是治病。（I：是不是医生跟你说这是遗传病？） 不是，医生不会这样说这是遗传病。就算知道这是遗传病也不会说的。这是医生的医德。（I：为什么医生道德就不会说？）医生不能跟病人说他的遗传病。医生只是治病的。好像你是教授就是教授，你放问我你就是访问我。他只是治疗某个人就是这个人，治疗某种病就是某种病。跟私人的事没有关。（I：这不算事私人的事。）医生就是只说这个病。他所负责的病。（I：医生有没有跟你说这是遗传病？）没有。（I：这是你自己觉得）是的。遗传病多半是从family tree 哪里知道的，不是医生说的。我相信80% 的医生都不会说的。

**Reference 2 - 7.27% Coverage**

I:您会和家庭医生讨论您的“家族病史”吗？

P：会的。（I：多久讨论一次？）我需要看病的时候就跟他说。（I：你多经常去看病？）（笑）早知道我就不接受你的访问，这么老套的问题。有没有人接受你的访问？（I： 你每次看医生你都会讨论一次？）不是，喉咙疼的时候会说，如果手脚疼就不会说了。（笑）（I：当你和医生说的时候，医生有没有建议你吃什么药或做什么检查？）这个没有，没有。（笑）（I：你上次跟医生讨论是什么时候？）三年前。

I: 您认为和您的家庭医生讨论您的“家族病史”的障碍是什么? 就是说，你看手脚的时候就不会说你的家族病史。

P：我不会跟他说。这是不需要的。（I： 三年前你跟医生说还是医生问你？）我跟医生说， 医生不会问的。（I：三年前的那一次是你自己跟他说的， 你为什么会跟他说？）因为我喉咙疼，我就跟他说了。（I：医生从来没有问过你详细的家族病史？像爸爸妈妈的病。）没有。（I：就是从来没有讨论过。因为没有详细的问过。）没有。他没有。（I：你从不和医生讨论是因为你觉得不重要？）这个不重要。（I：你并没有觉得不舒服去跟医生讨论？）是，如果不舒服 还是会跟医生讨论。（笑）。

**<Participant # 14. > - § 1 reference coded [1.84% Coverage]**

**Reference 1 - 1.84% Coverage**

（I：为什么不主动跟医生说？）唉，你也知道中国人，除非是熟人，聊起来就聊。家丑不外扬了。有什么好说的？事实上也没有必要。我也就懒得说了。何必把这种事到处宣扬。也不是好事。即使是好事，也没有必要吹牛。

**< Participant #24. > - § 1 reference coded [1.60% Coverage]**

**Reference 1 - 1.60% Coverage**

I: 是不是有时跟他说，有时不跟他说。

P: 应该是这样的。因为，怎么说，这个也不关他的事情。

I: 所以就没有每次都跟他说，是不是？

P: 嗯

**< Participant #38 > - § 1 reference coded [1.49% Coverage]**

**Reference 1 - 1.49% Coverage**

I: 好啊，那下一个就是想问问，就是和您的家庭医生讨论这个家族病史的障碍是什么？有没有这方面的顾虑呢？

P: 很大的顾虑没有。如果有必要的话，我会毫不保留地去说。但是，当然不可避免多多少少还是有一些心里障碍啦。
