# Supplementary material for: Chinese Americans’ Views and Use of Family Health History: A Qualitative Study
Source: PLoS One. 2016 Sep 20;11(9):e0162706. doi: 10.1371/journal.pone.0162706 (PMC5029932; doi:10.1371/journal.pone.0162706)
Supplement: S1 File — (ZIP) [file pone.0162706.s001.zip › Data/Barriers to discuss with doctors/Lack of FHH collection.docx]

**Name:** Lack of FHH collection

**<Participant #18. > - § 1 reference coded [2.22% Coverage]**

**Reference 1 - 2.22% Coverage**

I ：那每次看医生都谈吗？

P: 他们问就谈，不问就不谈。其实本身我了解的也不多。

I ：主要的你没有跟医生谈是因为他们不问，你也不说？

P: 应该是吧，小病么。

I: 那么你说你自己了解的不多，也是这个原因？

P: 对。

**<Participant #23. > - § 1 reference coded [1.28% Coverage]**

**Reference 1 - 1.28% Coverage**

I: 那你为什么没有跟你的一生讨论你的家族病史呢？

P: 因为我对自己家族的病史了解不深，所以就没有讨论。

**<Participant #39 > - § 1 reference coded [1.98% Coverage]**

**Reference 1 - 1.98% Coverage**

I: 那就是您认为和家庭医生讨论家族病史有没有什么障碍？

P: 我倒觉得不会，没有什么障碍。关键就是看我自己知道多少。有些信息不知道。

I:也就是说你想说，但不知道说什么，没有这方面的信息提供。但如果有信息，不会介意进行交流。是这样么？

P: 对。

**<Participant #48 > - § 1 reference coded [1.90% Coverage]**

**Reference 1 - 1.90% Coverage**

I:所以你认为，你和你的家庭医生讨论你的家族病史的障碍是什么？你说你不知道，所以你就没有办法讨论。

P:我想一方面是我不知道，另外一方面是医生也没有特别关注这个。我是说中国的医生。美国的医生，他也只是从你口中传，他怎么去确定呢，他不知道啊！所以说这很难说。比如说我弟弟青光眼这个事情，它是遗传么？那你妈妈有么？我妈妈有。那我妈妈有，我弟弟有，但我没有啊，再说你妈妈上面有没有我不知道。他怎么确定它是遗传呢？他不知道。他不可能通过两例去确定是遗传吧。最多写个可疑性遗传。
