# Supplementary material for: Chinese Americans’ Views and Use of Family Health History: A Qualitative Study
Source: PLoS One. 2016 Sep 20;11(9):e0162706. doi: 10.1371/journal.pone.0162706 (PMC5029932; doi:10.1371/journal.pone.0162706)
Supplement: S1 File — (ZIP) [file pone.0162706.s001.zip › Data/Barriers to discuss with doctors/Lack of inquiries from U.S. doctors .docx]

**Name:** Lack of inquiries from U.S. doctors

**<Participant #03. > - § 1 reference coded [0.56% Coverage]**

**Reference 1 - 0.56% Coverage**

P： 哦，有，我跟你讲，在美国的医生，她不会主动要我去检查那些东西，都是我跟她说我要检查这个这个，然后我有漏掉的，他就没有检查。

**< Participant #04 > - § 1 reference coded [1.32% Coverage]**

**Reference 1 - 1.32% Coverage**

I: 那你有没有和家庭医生讨论过家族病史呢？

P: 没有。

I：为什么？

P: 一来呢，他们就是问一下，因为没有很明显的家庭病史，所以就没有讨论。

I: 他也就是稍微提一下？

P: 对。

**< Participant #07. > - § 2 references coded [1.43% Coverage]**

**References 1-2 - 1.43% Coverage**

I: 那你为什么不跟你的家庭医生讨论你的家族病史？

P: 因为医生没有问，就不会去讨论那。

I: 所以你认为你在美国看病，医生都不会问你的家族病史，所以你不会跟他去讨论。

P: 美国医生？对啊，之前看病都没有问过啊。

**< Participant #11. > - § 1 reference coded [0.91% Coverage]**

**Reference 1 - 0.91% Coverage**

（I：医生从来没有问过你详细的家族病史？像爸爸妈妈的病。）**P:** 没有。（I：就是从来没有讨论过。因为没有详细的问过。）**P:** 没有。他没有。

**< Participant #14. > - § 1 reference coded [0.86% Coverage]**

**Reference 1 - 0.86% Coverage**

I:您会和家庭医生讨论您的“家族病史”吗？多久讨论一次？

Ｐ：没有，他没有问。我也没提。

**< Participant #16 > - § 1 reference coded [1.20% Coverage]**

**Reference 1 - 1.20% Coverage**

I:您认为和您的家庭医生讨论您的“家族病史”的障碍是什么?

P：他没有要求。

**< Participant #17. > - § 1 reference coded [2.81% Coverage]**

**Reference 1 - 2.81% Coverage**

I:您会和家庭医生讨论您的“家族病史”吗？

P：医生根本不会问。美国的医生也没有问。根本是我看医生，所以在表格上也只是问我自己的病史，没有问家族其他人的。（I：医生没问，你也不说？）因为是我看医生，医生也只是问我本身的是，从来不问其他的。

**< Participant #18. > - § 1 reference coded [1.33% Coverage]**

**Reference 1 - 1.33% Coverage**

I ：那每次看医生都谈吗？

P: 他们问就谈，不问就不谈。其实本身我了解的也不多。

I ：主要的你没有跟医生谈是因为他们不问，你也不说？

**< Participant #19. > - § 1 reference coded [1.19% Coverage]**

**Reference 1 - 1.19% Coverage**

P: 如果你有什么病，医生只会开些药给你啦。

I: 就是说你的医生没有问你。

P:没有哇。

**< Participant #22 > - § 1 reference coded [2.22% Coverage]**

**Reference 1 - 2.22% Coverage**

I: What are the barriers to discuss your family health history information with your health care providers?

P: I don’t go to the doctor, I go to the women check-up, and also they won’t really ask me.

**< Participant #23 > - § 1 reference coded [2.04% Coverage]**

**Reference 1 - 2.04% Coverage**

I: 那你有没有和家庭医生讨论过家族病史呢？

P: 没有。

I: 从来没有么？

P: 没有。

I：你去看医生，医生没有问你么？

P: 只是简单地说说而已。

**< Participant #25. > - § 2 references coded [3.59% Coverage]**

**References 1-2 - 3.59% Coverage**

I: 那么有没有跟你的医生讨论过家族病史呢？

P: 没有。

I: 从来都没有？

P: 没有。因为去看病都是些小毛病。

I: 那你觉得没有和医生讨论的原因是什么呢？

P 我觉得这个是医生尊重病人的隐私吧，你愿意讲，人家就愿意听吧。

I: 那你为什么没有问呢？

P: 因为那个时候也不知道奶奶是怎么死的。

I: 或者说你们家没有很明显的遗传病。

P: 没有。

**< Participant #26. > - § 2 references coded [5.53% Coverage]**

**Reference 1 - 1.13% Coverage**

I: 但是就是说你的family doctor没有问过你的？那你的family doctor从来都没有跟你说过这个有关家族病史的情况？

P: 没有。

**Reference 2 - 4.39% Coverage**

I: 为什么他不问，你觉得？或者说为什么你没有主动给他们说呢？譬如看见医生就说医生我们家族有什么什么疾病？为什么不这样做呢？

P: 嗯，可能有，因为，又一次他叫我查胆固醇。他问过我any family member has cholesterol, 但是他没有讲到什么遗传病。不过，他就已经叫我every two years去检查一下了, 他还叫我去prevent heart disease, 可能他有这个。然后，可能是，真的是 regular procedure，但是做这个procedure的时候他也就问。

I: 也就是他没有特别地去问到有关这个家族史的问题。

P: 我觉得没有。

**< Participant #28. > - § 1 reference coded [1.75% Coverage]**

**Reference 1 - 1.75% Coverage**

I:因为不常常看医生， 不能常跟医生说。

P：通常第一次去看医生的时候会看家族病史会仔细一点，再去看的时候， 或是身体检查时没有什么事，譬如检查子宫没有事，他（医生）就不会再和你提这些事（家族病史）。

**< Participant #29. > - § 2 references coded [5.81% Coverage]**

**References 1-2 - 5.81% Coverage**

I:您会和家庭医生讨论您的“家族病史”吗？

Ｐ：我都说了，我见医生的机会很少。（上次看医生时什么时候？）一年前吧，（I：那次看医生有没有谈到家族病史？）（Ｐ摇头）。上一次谈但家族病史的是什么时候？）大概是四，五年前。是一个身体检查，医生发现我的胆固醇偏高，但还没到要吃药控制的时候。他没问，我也没有主动跟他说。（I：其实那次也没有谈到家族病史。那是什么时候跟医生说过（家族病史）？）大概是十年前了。

I:您认为和您的家庭医生讨论您的“家族病史”的障碍是什么?

Ｐ：也许他（医生）认为不是问题，高血压在现在也不是什么大不了的病。他也没所谓。当他告诉我胆固醇偏高时，我跟他说我的家族有高血压。他可能也知道即使是医生本人，到年纪大了，也会有这种问题。这是很正常，

**< Participant #33. > - § 1 reference coded [3.26% Coverage]**

**Reference 1 - 3.26% Coverage**

I：您会和您的家庭医生讨论您的“家族病史”吗？多久讨论一次？

P：没有讨论过，医生也不会问。我最近看家庭医生是几个月前参加体检，也就是化验血呀，称个体重什么的，自己也没有什么病，所以医生也没有多问；除非有高血压和心脏病史，一般查体都不会涉及到“家族病史”。

**< Participant #36 > - § 1 reference coded [1.06% Coverage]**

**Reference 1 - 1.06% Coverage**

I: 那有没有和家庭医生讨论过家族病史呢？

P:没有讨论过。

I: 没有讨论过，是吧？！他也没有问过您么？

P: 没有。

**< Participant #44 > - § 1 reference coded [3.45% Coverage]**

**Reference 1 - 3.45% Coverage**

I: 那有没有和家庭医生一块讨论过家族病史啊？

P: 嗯，会得，如果有这种机会，要是，要需要讨论的话，我是比较乐意谈的。

I: 但是目前为止 有没有讨论过。

P: 到目前为止，没有讨论过。

I: 如果需要和医生讨论的话，你觉得会不会有什么障碍？

P: 嗯，我认为没什么障碍。我觉得，嗯，对我自己来说，我是比较乐意讨论的。没有什么心理或者什么障碍。

**< Participant #45. > - § 1 reference coded [1.86% Coverage]**

**Reference 1 - 1.86% Coverage**

I: 那原因是？

P: 嗯，没有场合需要讨论啊，而且医生也没有要求正式跟我讨论啊。只是去看病的时候，会填一个什么表格，这里面好像提到过家族病，呵呵，我也记不清楚了。嗯，我觉得，还是一个意识问题。没觉得这个东西有多迫切，多正式。

**< Participant #47 > - § 1 reference coded [3.69% Coverage]**

**Reference 1 - 3.69% Coverage**

I: 那么，有没有和您的医生讨论过这个家族病史么？

P 嗯。。。看病的时候要填一个像是questionnaire之类的表格。

I: 那有没有具体地去讨论过呢？

P: 讨论啊？！。。。。那没有。只是说填写了表，他知道一些information。

I: 那大概多久一次呢？

P: 就是和看病的频率是一样的。

I: 那您认为和家庭医生讨论家族病史会不会有一些障碍？

P: 应该没有吧。我们对家庭医生也应该是很trust的。
