# Supplementary material for: Chinese Americans’ Views and Use of Family Health History: A Qualitative Study
Source: PLoS One. 2016 Sep 20;11(9):e0162706. doi: 10.1371/journal.pone.0162706 (PMC5029932; doi:10.1371/journal.pone.0162706)
Supplement: S1 File — (ZIP) [file pone.0162706.s001.zip › Data/Barriers to discuss with doctors/Lack of trust with U.S. doctors.docx]

**Name:** Lack of trust with U.S. doctors

<**Participant #**32. Participant_2> - § 1 reference coded [4.03% Coverage]

Reference 1 - 4.03% Coverage

P：聊也没怎么聊，我觉得医生不care about your health, they don’t 。不能太rely on doctors。你是他（她）的patient ,你有病的时候才去找医生。而且你真去找医生有没有solutions 也不知道。

I：下一个问题您认为和您的家庭医生讨论您的“家族病史”的障碍是什么呢？

I：她刚才有讲她的医生根本不care 她的健康，也没有时间去和她讨论。

P：医生没有时间跟我讨论，如果他给我时间我当然愿意和他讨论了。

I：所以你觉得医生根本没有时间，也不care.那你说的是美国的医生还是ancient的医生？

P：我以前的保险是白人医生，每次他都匆匆忙忙的，实际上大部分的时间都是护士给你量量或吃药，我已经有好几年不吃药了。我都是锻炼哪，注意饮食健康啊，take supplement ,take good vitamin, good nutrition。
