# Supplementary material for: Chinese Americans’ Views and Use of Family Health History: A Qualitative Study
Source: PLoS One. 2016 Sep 20;11(9):e0162706. doi: 10.1371/journal.pone.0162706 (PMC5029932; doi:10.1371/journal.pone.0162706)
Supplement: S1 File — (ZIP) [file pone.0162706.s001.zip › Data/Barriers to discuss with doctors/Language Barrier.docx]

**Name:** Language Barrier

**<Participant #30. > - § 1 reference coded [2.27% Coverage]**

**Reference 1 - 2.27% Coverage**

I: 您会和家庭医生讨论您的“家族病史”吗？

P：没有。

I: 您认为和您的家庭医生讨论您的“家族病史”的障碍是什么?

P：我没有家庭医生。还有语言的问题。很多医学词语（英语）我都不懂， 语言障碍。

**<Participant #43. > - § 1 reference coded [1.49% Coverage]**

**Reference 1 - 1.49% Coverage**

I: 那您认为和您的家庭医生讨论这个家族病史有没有什么障碍么？

P：没有什么障碍，因为他是医生么。除非是有时候讲不出来，比如说语言的问题。因为他是美国医生么，所以有些病我们不会讲。
