# Supplementary material for: Chinese Americans’ Views and Use of Family Health History: A Qualitative Study
Source: PLoS One. 2016 Sep 20;11(9):e0162706. doi: 10.1371/journal.pone.0162706 (PMC5029932; doi:10.1371/journal.pone.0162706)
Supplement: S1 File — (ZIP) [file pone.0162706.s001.zip › Data/Barriers to discuss with doctors/Never or rarely visiting doctors in the U.S. .docx]

**Name:** Never or rarely visiting doctors in the U.S.

**<Participant #05. > - § 1 reference coded [0.47% Coverage]**

**Reference 1 - 0.47% Coverage**

I: 为什么呢？

P: 为什么，因为我很少看医生啊。我就只有看过两次而已。

**< Participant #06. > - § 2 references coded [1.96% Coverage]**

**Reference 1 - 0.62% Coverage**

I: 你为什么不在跟美国的家庭医生讨论呢？

P: 因为我又不是不要去看病，看病又不是不要花钱。然后还要约时间啊，哪有美国时间啊。

**Reference 2 - 1.34% Coverage**

P: 对啊。

I: 在台湾也要花钱嘛！

P: 花得钱少多了。你说在大医院，你说花的多，了不起十几块美金而已么。对不对？最高才十几块美金，而且还包括检查也。我去那儿半年检查一次，花不到三十块美金。而且没有必要在美国啊，我每半年回去一次，我就不需要啦。除非是我，希望上帝不要让我在这里生病啦。

**< Participant #12. > - § 1 reference coded [3.37% Coverage]**

**Reference 1 - 3.37% Coverage**

I:您会和家庭医生讨论您的“家族病史”吗？

P：有的有的。（I：多久讨论一次？每次看医生时谈到？）是的。我很少看医生，只是不舒服是看一下医生。（I：你多经常看医生？）我很小看医生，我很注重保养。而且现在每年都做一次身体检查，查查心跳什么的。

**< Participant #22 > - § 1 reference coded [4.08% Coverage]**

**Reference 1 - 4.08% Coverage**

I: How often have you discussed your family health history information with your health care providers?

P: I don’t really talk about my family health history with my doctor. Sometimes he just tells me to fill out paperwork, and he would ask me by the way. (I: But he won’t ask you about it?) No, I don’t go to a doctor here. I go to a women check-up.

**< Participant #28. > - § 1 reference coded [2.68% Coverage]**

**Reference 1 - 2.68% Coverage**

I：为什么不常常跟医生说？什么是讨论“家族病史”的障碍？

P：一来少看医生，二来（医生）有一个家族病史的LIST跟进。譬如我有胆固醇高的问题。她会跟我说因为我的家族中有不少人有心脏病，爷爷和爸爸都有心脏病，而且因此而去世的，家族历史由此遗传。所以我必须吃DIET的东西，而且要运动。因为家族历史有此遗传。

**< Participant #29 > - § 1 reference coded [1.02% Coverage]**

**Reference 1 - 1.02% Coverage**

I:您会和家庭医生讨论您的“家族病史”吗？

Ｐ：我都说了，我见医生的机会很少。（上次看医生时什么时候？）一年前吧，

**< Participant #31. > - § 1 reference coded [0.27% Coverage]**

**Reference 1 - 0.27% Coverage**

I：那你在美国看过病吗？

P：我没有。

**< Participant #42 > - § 1 reference coded [3.17% Coverage]**

**Reference 1 - 3.17% Coverage**

I: 那么，您有没有和医生讨论过这个家族病史？

P: 很少。去看病的时候才提到说是，家中有没有高血压？

I： 也就是去看病的时候才有可能提到。所以也就和你看病的频率差不多，一年一次的样子？

P: 对。

I: 那和医生讨论这方面，有没有什么障碍？

P: 没有。

**< Participant #46> - § 1 reference coded [1.90% Coverage]**

**Reference 1 - 1.90% Coverage**

I: 那有没有和家庭医生讨论过？

P: 没有，因为我没有家庭医生。

I: 那原因是？

P: 我给你说，这个和个人的健康很有关系。因为没有什么很大的疾病。比如说我母亲有高血压，你还没有得这个病，所以你可能没有机会去讨论。一旦有得了这个病，你可能就会去讨论。
