# Supplementary material for: Chinese Americans’ Views and Use of Family Health History: A Qualitative Study
Source: PLoS One. 2016 Sep 20;11(9):e0162706. doi: 10.1371/journal.pone.0162706 (PMC5029932; doi:10.1371/journal.pone.0162706)
Supplement: S1 File — (ZIP) [file pone.0162706.s001.zip › Data/Barriers to discuss with doctors/No Barriers.docx]

**Name:** No Barriers

**<Participant #02. > - § 1 reference coded [0.88% Coverage]**

**Reference 1 - 0.88% Coverage**

I: 那你有没有任何跟你的家庭医生讨论你的家族病史障碍么？

P: 没有问题。我一定要跟他讲，因为我跟他讲了，他才会了解。这个不要隐瞒，因为他才知道，他才会帮助我。

**< Participant #08. > - § 1 reference coded [0.51% Coverage]**

**Reference 1 - 0.51% Coverage**

I: 所以，你有没有跟你的家庭医生讨论家族病史的障碍？

P: 还好啊，她是女孩子，有时候跟她讲话还蛮那个，没什么问题。

**< Participant #20. > - § 1 reference coded [2.39% Coverage]**

**Reference 1 - 2.39% Coverage**

I: 你觉得有没有什么不好意思给医生说你的家族病史呢？

P: 这个我理解。有些人有精神病啊，他都不敢讲。

I: 我妈就说家里有什么高血压啊，你都不要讲，不然你就要嫁不出去。

P: 他们早就说过有这个病么，我现在才找到。

I: 所以你就是什么都可以跟医生讲，一点都没有不好意思。

I: 医生是美国人么？白人。

P: 白人。

I: 你有多相信你的医生？

P: 我相信他啊。

**< Participant #27. > - § 1 reference coded [0.43% Coverage]**

**Reference 1 - 0.43% Coverage**

I: 就是你跟他谈的时候有没有什么障碍。

P: 没有。

**< Participant #35. > - § 2 references coded [3.69% Coverage]**

**Reference 1 - 2.48% Coverage**

I: 那下一个想问问您，什么是您向您的家庭医生提供准确的家族病史的，这个信息的障碍呢？有没有这种障碍。

P: 我还是可以提供比较精确的家族病史。因为我的病史里面并没有什么私自的东西。所以说很自然的就沟通。

I: 无所谓有什么隐私的这一部分。

P: 我的家庭医生是中国人。如果有需要隐私的话，我可能会找美国的家庭医生。所以说，不管怎么说我都会向他们提供的。

**Reference 2 - 1.21% Coverage**

I: 那和家庭医生有没有讨论家族病史呢？

P:不会特意去讨论，他要问我生了什么病啊，或者哪里不舒服，他就再问你家里有没有这种病史的话，我就如实地告诉他。比如说高血压，他就会问。

**< Participant #37. > - § 2 references coded [3.96% Coverage]**

**Reference 1 - 2.37% Coverage**

I: 明白。好，下一个问题是想问一下，如果是您搜集过家族病史相关的的信息，为什么您会这么做？如果您从未搜集过，也想问一下原因是什么呢？

P: 我想我要是想去搜集的话，我对自己的健康产生了一些怀疑，或者怎么样子，对我自己的健康关心的话，我可能多去了解一下。

I: 那您自己去了解过么？

P: 自己还没有。呵呵。

**Reference 2 - 1.59% Coverage**

I: 那您觉得您和您的家庭医生讨论这个家族病史的障碍是什么？有么？有没有这个障碍。

P: 对我来说，我觉得应该很好。因为我跟我的家庭医生的关系都还不错，然后又都是女医生，没有什么。什么都可以说，应该没有什么障碍。

**< Participant #40 > - § 1 reference coded [0.46% Coverage]**

**Reference 1 - 0.46% Coverage**

I: 那就是有没有什么障碍？

P: 没有。

**< Participant #41 > - § 2 references coded [0.51% Coverage]**

**References 1-2 - 0.51% Coverage**

I: 那这个和家庭医生讨论有没有什么障碍？

P: 没有。
