# Supplementary material for: Chinese Americans’ Views and Use of Family Health History: A Qualitative Study
Source: PLoS One. 2016 Sep 20;11(9):e0162706. doi: 10.1371/journal.pone.0162706 (PMC5029932; doi:10.1371/journal.pone.0162706)
Supplement: S1 File — (ZIP) [file pone.0162706.s001.zip › Data/Barriers to discuss with doctors/Perceived no importance in discussing FHH with doctors.docx]

**Name:** Perceived no importance in discussing FHH with doctors

**<Participant #09. > - § 2 references coded [2.99% Coverage]**

**References 1-2 - 2.99% Coverage**

I: 那有没有什么讨论呢？

P: 我就会告诉他说我爸爸有cancer，然后就去做相应的检查。

I: 所以你就没有跟任何家庭医生讨论家族病史。

P: 没有，我就跟他讲。现在基本上每年我都测测，他说没有事，就没事了。

I: 为什么不跟你的家庭医生讨论过家族病史吗？有什么障碍么？

P: yeah。不会想到去。

**<Participant #11. > - § 1 reference coded [1.91% Coverage]**

**Reference 1 - 1.91% Coverage**

（I：医生从来没有问过你详细的家族病史？像爸爸妈妈的病。）**P:**没有。（I：就是从来没有讨论过。因为没有详细的问过。）**P:**没有。他没有。（I：你从不和医生讨论是因为你觉得不重要？）**P:**这个不重要。（I：你并没有觉得不舒服去跟医生讨论？）**P:** 是，如果不舒服 还是会跟医生讨论。（笑）。

**<Participant #15. > - § 1 reference coded [3.43% Coverage]**

**Reference 1 - 3.43% Coverage**

I: 您会和家庭医生讨论您的“家族病史”吗？多久讨论一次？

P: 有的，我又跟医生说，引文我们家有这个病，我有点害怕，看医生时就跟她说了。（I：你只是第一次看医生是跟他说？）是的。（I：只说过一次？）是啊。这个病只说过一次，医生就会知道了。就不用说了。我妈有肠癌，我没有检查过肠，我很害怕，所以去年我也去了做检验。没事就安心了。

**<Participant #21. > - § 2 references coded [2.98% Coverage]**

**Reference 1 - 2.11% Coverage**

P: 就是我去看病啊，就告诉他。一般医生都会问你们家里有什么那个病史。

I: 那么只是说过一次，还是常常会提到？

P: 没有。就是一开始。现在我发现在美国如果去看医生的话，一开始就会问你一下有没有家族的病史，然后你告诉他，可能他会记下来，什么的我就不清楚了。

**Reference 2 - 0.87% Coverage**

I: 那没有常常给医生提，主要的障碍是什么呢？

P: 我觉得没什么必要再跟他提，反正他已经知道了么。

**<Participant #34. > - § 1 reference coded [1.01% Coverage]**

**Reference 1 - 1.01% Coverage**

I：那您和您的家庭医生经常讨论您的家族病史吗？

P：家族病史讨论一次就可以呀，不需要经常讨论的。

**<Participant #45. > - § 1 reference coded [1.86% Coverage]**

**Reference 1 - 1.86% Coverage**

I: 那原因是？

P: 嗯，没有场合需要讨论啊，而且医生也没有要求正式跟我讨论啊。只是去看病的时候，会填一个什么表格，这里面好像提到过家族病，呵呵，我也记不清楚了。嗯，我觉得，还是一个意识问题。没觉得这个东西有多迫切，多正式。
