# Supplementary material for: Chinese Americans’ Views and Use of Family Health History: A Qualitative Study
Source: PLoS One. 2016 Sep 20;11(9):e0162706. doi: 10.1371/journal.pone.0162706 (PMC5029932; doi:10.1371/journal.pone.0162706)
Supplement: S1 File — (ZIP) [file pone.0162706.s001.zip › Data/Barriers to discuss with doctors/Self-claimed healthy family.docx]

**Name:** Self-claimed “healthy family”

**<Participant #01. > - § 1 reference coded [2.61% Coverage]**

**Reference 1 - 2.61% Coverage**

I:您会和家庭医生讨论您的“家族病史”吗？

P: 没有。医生有问过。但我基本上都是no. ( I: 那你基本上没有跟医生讨论过？) 没有，只有在问病历会问到父母有没有疾病。(I:为什么不主动跟医生讨论？）我自己觉得还蛮健康的。就没有特别的问。

（I:你有兄弟姐妹吗？）两个哥哥和一个弟弟。他们都蛮健康的。他们都住在美国。（I: 他们有高血压之类？）没有，都没有高血压。我的那个（心律不齐）不是遗传的。

**<Participant #04. > - § 1 reference coded [0.56% Coverage]**

**Reference 1 - 0.56% Coverage**

I：为什么？

P: 一来呢，他们就是问一下，因为没有很明显的家庭病史，所以就没有讨论。

**<Participant #10. > - § 1 reference coded [2.23% Coverage]**

**Reference 1 - 2.23% Coverage**

I: 所以你就没有跟任何家庭医生讨论家族病史。

P: 没有，我就跟他讲。现在基本上每年我都测测我的基因，他说没有事，就没事了。

I: 为什么不跟你的家庭医生讨论过家族病史吗？有什么障碍么？

P: 因为没有什么好讲的。

I: 所以就是觉得自己没有什么生病，所以就没讲。

**<Participant #13. > - § 1 reference coded [2.53% Coverage]**

**Reference 1 - 2.53% Coverage**

I:您认为和您的家庭医生讨论您的“家族病史”的障碍是什么?

Ｐ：我们都没有什么很严重的病出现，所以不用说了。不过到严重了，或者老了，还是会跟医生说的。我们现在还小。而且我们会死于不同方式。（I：会有其他的原因导致死去，不单是遗传病？）是的，好像抽烟。

**<Participant #25. > - § 1 reference coded [1.28% Coverage]**

**Reference 1 - 1.28% Coverage**

I: 那你为什么没有问呢？

P: 因为那个时候也不知道奶奶是怎么死的。

I: 或者说你们家没有很明显的遗传病。

P: 没有。

**<Participant #46 > - § 1 reference coded [1.90% Coverage]**

**Reference 1 - 1.90% Coverage**

I: 那有没有和家庭医生讨论过？

P: 没有，因为我没有家庭医生。

I: 那原因是？

P: 我给你说，这个和个人的健康很有关系。因为没有什么很大的疾病。比如说我母亲有高血压，你还没有得这个病，所以你可能没有机会去讨论。一旦有得了这个病，你可能就会去讨论。

**<Participant #49 > - § 1 reference coded [1.98% Coverage]**

**Reference 1 - 1.98% Coverage**

I: 那你从来没有跟你的家庭医生讨论过你的家族病史。

P: 没有。因为我现在没有什么状况，他给我检查，都很好，没事。而且我每次产检，都在量血压，都很好，没有问题。

I: 所以你认为，你和你的家庭医生讨论你的家族病史的障碍是什么？

P: 不会。我一般，我都会告诉他。填表格的时候我都会写上。
