# Supplementary material for: Chinese Americans’ Views and Use of Family Health History: A Qualitative Study
Source: PLoS One. 2016 Sep 20;11(9):e0162706. doi: 10.1371/journal.pone.0162706 (PMC5029932; doi:10.1371/journal.pone.0162706)
Supplement: S1 File — (ZIP) [file pone.0162706.s001.zip › Data/Barriers to discuss with family members/Chinese cultural taboo and stigma_043015.docx]

**Name:** Chinese cultural taboo and stigma

<Participant # 07. > - § 2 references coded [7.86% Coverage]

Reference 1 - 6.20% Coverage

P: 主要就是不熟吧，然后有些事情也不一定会跟你讲。

I： 为什么疾病的事情不会跟你讲？

P: 主要是不熟吧，所以有些疾病的事情就不会跟你讲。

I: 譬如说，糖尿病，亲戚就不会跟你讲。

P: 糖尿病，还好啦，因为是长期的病。

I: 那什么病他不会跟你讲？

P: 其实，我也不是很清楚。像癌症，应该会给你讲吧。但是一些奇怪的病，像你了肺结核啊，艾滋病啊，那种，不适合被人家知道的病，通常人家不会告诉你。

I: 像艾滋病啊，肺结核啊。

P: 对，一般都比较不会啦，但糖尿病，癌症啊，一些都可以得到一些资讯啦。

I：okay，那你认为像那种mental disease呢？

P: 通常不会告诉你啦。

I: 为什么精神病就不会跟你讲。

P: 因为亚洲人呐，就不会给你讲。

I：为什么亚洲人就不会讲。

P： 因为文化啊，得精神病是一种耻辱啊，没有人会告诉你。

I: 所以这是文化，okay。是所有得精神病么？还是？

P: 嗯，现在应该开放一点了，有一些像忧郁症，或者睡眠不好，通常有时候有些人会知道一些，但大部分，都还是不愿意说出来。

Reference 2 - 1.66% Coverage

I: 为什么你没有跟你的家族讨论过？

P: 因为这不是什么讨论重点。

I: 什么意思？

P: 因为你不会常常去问人家生病了没有。你会得什么病。通常是人家得了什么病，你听说。不会去直接去问说，最近，有谁生病了，是不是有谁得癌症啊，什么，没有人会去这样问。

<Participant # 16. > - § 2 references coded [5.26% Coverage]

Reference 1 - 2.23% Coverage

I:你说重要，那您有没有收集过您的“家族病史”信息？（注：家族病史表）

P：没有。（I：为什么不去收集？）P:（笑）为什么不？没有老想着这种事。没有机会去收集。

Reference 2 - 3.03% Coverage

I:您有没有和您的家庭一起讨论您的“家族病史”么？多久讨论一次？

P：没有。（I：你既然知道你的家族病史，为什么不讨论一下？）P：这也只是想，猜的，谁谁谁有某种病，没有很明确知道，而且，医学上的事，也说不清。

<Participant # 17.> - § 2 references coded [3.13% Coverage]

Reference 1 - 1.39% Coverage

I:您认为收集“家族病史”的相关信息重要吗？

P：很重要。但我没有时间去做这个事。（I：你没有收集过。）**P:**没有。

Reference 2 - 1.74% Coverage

（I：为什么不谈及呢？）**P:**他们不说，我没话可说。你的问题很难答。（I：没关系，你不想说你就说不想说，你不知道就说不知道。）**P:**他们（父母）不说，我又没问。

<Participant # 18.> - § 2 references coded [2.60% Coverage]

References 1-2 - 2.60% Coverage

P: 很久，大概一两年。通常我们都很少看医生，因为都没有什么病，所以也不会搬出来讨论。而且我跟父母也没住在一起。然后没什么大病啊，也不会在打电话的时候搬出来讲吧。报喜不报忧嘛。通话的时候如果是小事情通常不会搬出来讲。

I: 也就是没讨论的原因主要是因为大家距离很远。

P: 没住在一起。

<Participant # 19. > - § 1 reference coded [5.80% Coverage]

Reference 1 - 5.80% Coverage

I: 那么你有没有去搜集一下你的家庭病史的信息？有没有去问一下？

P: 没有。

I: 为什么没有去做？

P: 因为很少有这种病。

I: 那么你是从哪里知道你家族病史的信息？

P: 当然是家里人告诉我，才知道。

I: 家里人是指？

P: 父母啊。

I: 你有没有和你的家里的人一起讨论家族病史？就是说我们家里人得过什么病，就是我们要特殊注意。

P: 因为没有发生过这些事情啊，所以也就没有讨论过。

I: 因为没有发生过，所以你觉得就没有必要讨论。

P: 我们家族里面很少讨论这种问题。

<Participant # 23. > - § 1 reference coded [0.55% Coverage]

Reference 1 - 0.55% Coverage

I: 原因是什么呢？

P: 因为我害怕。

<Participant #30.> - § 1 reference coded [3.13% Coverage]

Reference 1 - 3.13% Coverage

I:您和您的家庭一起讨论您的“家族病史”么？

Ｐ：没有，（I：从没有？）没有，因为没有提到这个话题。（I：你爸从没提到他的爸妈是怎么死的？）没有。怎会提到一个死了二十多年的人？

I:您认为和您的家庭讨论您的“家族病史”的障碍是什么?

P：以前太小。现在又觉得没有必要。

<Participant #43> - § 1 reference coded [3.38% Coverage]

Reference 1 - 3.38% Coverage

P: 嗯。第一点，因为我们年纪还不到么。那第二点是，台湾的情况还不太一样。因为这些都是已经发生的，都是在台湾，而且我已经来这儿这么就了，和那边已经脱节了。那第三点就是，当时在台湾的时候，因为不是大房子，所以大家都分开住。分开住就会把亲戚这种亲密的关系疏远一点。这也有影响。所以，如果父母不提的话，我们也不会知道。那有时候，父母也觉得没有必要提，因为父母也觉得没有必要把病痛的东西让小孩子知道。这也是习惯啊，我们中国人的风俗习惯。

<Participant #47 > - § 1 reference coded [2.45% Coverage]

Reference 1 - 2.45% Coverage

I: 然后，如果没有收集过这方面的信息，那障碍是什么呢？

P: 嗯。。。一方面，文化上面就是有点忌讳，反正，就是死的么，就是。另外，可能自己也不是说，因为自己，嗯。。。也挺健康的。健康好像现在不是说特别重视吧。可能，一般都是，嗯。。。身体不好了，可能就比较重视自己的健康了，或了解这方面的东西。
