# Supplementary material for: Chinese Americans’ Views and Use of Family Health History: A Qualitative Study
Source: PLoS One. 2016 Sep 20;11(9):e0162706. doi: 10.1371/journal.pone.0162706 (PMC5029932; doi:10.1371/journal.pone.0162706)
Supplement: S1 File — (ZIP) [file pone.0162706.s001.zip › Data/Barriers to discuss with family members/No barriers.docx]

**Name:** No barriers

**<Participant # 05. > - § 1 reference coded [0.47% Coverage]**

**Reference 1 - 0.47% Coverage**

I: 你有没有跟你姑姑，爸爸妈妈讨论家族病史的障碍？

P: 障碍，没有也。

**< Participant # 06. > - § 1 reference coded [0.32% Coverage]**

**Reference 1 - 0.32% Coverage**

I: 所以说都没有什么障碍？

P: 你要讲可以啊，我也讲啊。对

**< Participant # 20. > - § 1 reference coded [0.79% Coverage]**

**Reference 1 - 0.79% Coverage**

I: 那你觉得有没有什么不方便？

P: 都没有，都没有不方便，就是哥哥姐姐，都可以，

I: 都没有什么障碍的，跟哥哥姐姐都可以说。

**< Participant # 22. > - § 1 reference coded [2.52% Coverage]**

**Reference 1 - 2.52% Coverage**

I:If you have collected your family health history information, why?

P: To just be aware of it. My father has diabetes, you see, and I might be able to get it too along with the heart disease, so I’m just being aware of this.

**< Participant # 27 > - § 1 reference coded [1.21% Coverage]**

**Reference 1 - 1.21% Coverage**

I: 也就是说你其实没有一个固定的时间要多久？反正什么时候看到了就会。

P: 对。

I: 为什么你天天给他们谈呢？

P: 因为家族史，你一旦有了这个病史，你并不能改变。

**< Participant # 31. > - § 1 reference coded [0.81% Coverage]**

**Reference 1 - 0.81% Coverage**

I：那如果你说经常，那是多久呢？

P：差不多每年谈个三，四次吧。

I：那你会不会觉得和你的家人讨论会有障碍？

P：没有。

**< Participant # 32. > - § 1 reference coded [1.28% Coverage]**

**Reference 1 - 1.28% Coverage**

I: 那你觉得您和您的家人讨论您的“家族病史”的障碍是什么？

P：我没有什么心理障碍。

I：但是你们会谈吗？就是有些人不愿意谈到父母有什么疾病。特别是mental 方面的问题他们会想提到。

P：没有，是因为我父母这几年身体都非常好，我这几年一直给他

**< Participant # 34. > - § 1 reference coded [0.77% Coverage]**

**Reference 1 - 0.77% Coverage**

I：那您和家人说起家族病时没什么障碍，是吗？

P：一点儿障碍都没有。

**< Participant # 35 > - § 1 reference coded [1.40% Coverage]**

**Reference 1 - 1.40% Coverage**

I: 那您觉得跟您的家庭讨论家族病史，有没有什么障碍没有？

P: 应该是没有，基本上，我没有主动去跟我的家庭医生去讨论。

I: 那和家庭呢？

P: 那没有。如果不是隐私病情的话，那就一般不会。

**< Participant # 36 > - § 1 reference coded [2.35% Coverage]**

**Reference 1 - 2.35% Coverage**

I: 好，下一个想问一下，什么是您向您的家庭医生提供精确的家族病史的信息的障碍呢？您个人有没有这方面的障碍？

P: 没有。我自己没有什么需要保密的理由啊，什么的。但有些信息，比如说祖父母这方面的，我觉得比较难去得到他们的状况。

I: 也就是说有时候不够准确，但是没有任何障碍，对吧？！

P: 对。

**< Participant # 37 > - § 1 reference coded [2.87% Coverage]**

**Reference 1 - 2.87% Coverage**

I: 好，下一个想问一下，什么是您向您的家庭医生提供精确的家族病史的信息的障碍呢？如果家庭医生问您有没有什么家族病史呢，您认为您会不会很hesitate去谈这个问题呢？

P: 嗯，我觉得对我来说可能还可以。可能对有些人，会有障碍。对我自己来说，我觉得都没有什么，因为是觉得，对自己有好处。如果医生问到的话，我会开诚布公地讲，把我知道的都说出来。

I: 哦，因为是医生么，所以可以讲出来。

**< Participant # 38. > - § 1 reference coded [1.86% Coverage]**

**Reference 1 - 1.86% Coverage**

I: 那您觉得跟您的家庭讨论家族病史的障碍是什么呢？

P: 基本上没有什么障碍。我和我的家人在讨论的时候，基本上没有什么障碍，只是说讨论的深度不是很够。因为像我的家庭的话，就我所知，没有什么很特别的家族病，或者怎么样，所以就缺乏一种，平时的这样一种很深入的交流的。

**< Participant # 40 > - § 1 reference coded [0.63% Coverage]**

**Reference 1 - 0.63% Coverage**

I: 那您认为和家庭讨论家族史的障碍是什么呢？

P: 没有什么障碍。

**< Participant # 41 > - § 1 reference coded [2.07% Coverage]**

**Reference 1 - 2.07% Coverage**

I: 那为什么这样，没有讨论过这个话题呢？

P：我们没有讨论，但是我们都知道。像我先生，你爸有糖尿病，有也要有糖尿病，你妈有胃病，你或许也会有胃病。

I: 那也就是说会在闲聊中，但是从来没有当做一个话题来讨论？

P: 没有，从来没有。

**< Participant # 47 > - § 1 reference coded [2.38% Coverage]**

**Reference 1 - 2.38% Coverage**

I: 针对你自己的家族情况，大概多久会讨论一次这个家族病史。有没有一个大概的频率。

P: 一年一次，或怎样。

I: 那么，谈论家族病史的障碍是什么呢？

P: 嗯。。。障碍估计也没有什么障碍，就是没有怎么重视吧。

I: 也就是说对家族病史这个课题的重视么？

P: 对。

**< Participant # 49 > - § 1 reference coded [0.63% Coverage]**

**Reference 1 - 0.63% Coverage**

I: 那你觉得和家庭讨论这个有什么障碍么？

P: 没有，我觉得我的父母都很开明，都可以讨论
