# Supplementary material for: Chinese Americans’ Views and Use of Family Health History: A Qualitative Study
Source: PLoS One. 2016 Sep 20;11(9):e0162706. doi: 10.1371/journal.pone.0162706 (PMC5029932; doi:10.1371/journal.pone.0162706)
Supplement: S1 File — (ZIP) [file pone.0162706.s001.zip › Data/Barriers to discuss with family members/Perceived insignificnace of discussing FHH with family members_043015.docx]

**Name:** Perceived insignificnace of discussing FHH with family members

<**Participant #** 02.> - § 2 references coded [2.92% Coverage]

Reference 1 - 1.51% Coverage

P: 对，还没有必要花这么多时间去搜集。

I: 还有么？

P: 就这样，我觉得还没有必要这样子。而且还有就是很多information，嗯，就是，如果我们有这方面的，都是医学上你可以得到控制，就是没有必要，好像没有这么urgent。或者是非做不可的。

I: 你是说医生会控制。

Reference 2 - 1.41% Coverage

I: 你有没有说从电视，或是报纸杂志看到说是，收集家族病史很重要。

P: 我没有听过。但我看报纸经常看到，这个是遗传病，这个是遗传有关。但没有说达到，说这个很重要，你要去了解。还没有看到过这样一个message这样写的。所以说有多重要，为什么有多重要我就不晓得。

<**Participant #** 07. > - § 3 references coded [5.35% Coverage]

Reference 1 - 1.81% Coverage

I: 好，那你有没有收集过家族病史的消息？

P: 嗯，没有啊，没有特地去收集。

I: 为什么？你有什么障碍么？为什么你不会去收集?

P: 因为收集了也不知道去做些什么？

I: 什么意思？

P: 也许听到过别人得了什么样的病，也不知道收集了会有什么样的用，对自己有什么用。

Reference 2 - 0.96% Coverage

I: 那你认为搜集家族病史重要么？

P: 嗯。。。讲起来似乎重要啦。但是没有，但是知道之后，也不知道专门，可以干些什么事情，或者说是对我有什么帮助。

Reference 3 - 2.57% Coverage

P: 因为你不会常常去问人家生病了没有。你会得什么病。通常是人家得了什么病，你听说。不会去直接去问说，最近，有谁生病了，是不是有谁得癌症啊，什么，没有人会去这样问。

I: 所以你的意思是说，你不会觉得这个东西很重要，然后觉得突然讨论这个，是么？

P: 嗯，也是啦。也不好开口随随便便就问人家，看到人家就说，你最近有没有身体不好？

I: 所以你觉得这不是日常生活的重点，没有那么重要。

P: 对。

<**Participant #** 30. > - § 1 reference coded [3.09% Coverage]

Reference 1 - 3.09% Coverage

I: 您和您的家庭一起讨论您的“家族病史”么？

Ｐ：没有，（I：从没有？）**P:**没有，因为没有提到这个话题。（I：你爸从没提到他的爸妈是怎么死的？）**P:**没有。怎会提到一个死了二十多年的人？

I: 您认为和您的家庭讨论您的“家族病史”的障碍是什么?

P：以前太小。现在又觉得没有必要。

<Participant # 39 > - § 1 reference coded [2.68% Coverage]

Reference 1 - 2.68% Coverage

I: okay，那么没有收集过这方面的信息的障碍是什么呢？

P: 就是说没有很正式地去做，就是，没有一个，没有一个，没有一个很重要的trigger。就是没有trigger，就是日常生活吧，可能没有突然想到这个病我是不是要去调查一下。没有这个意识。

I: Okay。还有没有其他原因。

P: 没有什么其他原因。关键是，对我来说还是因为没有trigger.

<**Participant #** 42. > - § 2 references coded [3.45% Coverage]

Reference 1 - 1.26% Coverage

I: 那您这边收集过家族病史的相关信息吧？就是说没有特意去搜集。

P: 嗯，没有。

I: 为什么呢？

Reference 2 - 2.19% Coverage

P：没有讨论的原因是，就是，就是，他如果说我得了，就得了，没有说讨论。

I: 是不是说对家族病史的idea比较淡，不会说是坐下来讨论。

P: 我也不知道。通常讲就是年纪大了。血压就会高。

<**Participant #** 44. > - § 2 references coded [7.19% Coverage]

Reference 1 - 5.60% Coverage

I: 那你觉得让你提供精确的家族病史的信息的话，那您觉得什么是你的障碍？

P: 障碍，嗯，就是，从小，没有，从小很少得到这方面的信息吧。

I: 嗯。

P: 就是父母也不大给我讲这些，而且医生，平常看比较regular的病，也没有。也可能和我家庭有关系吧。

I: 嗯。

P: 主要是没有这方面的信息吧。

I: 那也就是你从来没有收集过这方面的信息，对吧？

P: 我没有收集过。

I: 那没有收集过的原因是什么呢？就是障碍是什么呢？

P: 嗯。。。

I: 像刚才说到的，来源于父母和医生的这方面的信息就比较少，那还有没有其他的？

P: 嗯，感觉上没有。

Reference 2 - 1.59% Coverage

I: 那是为什么没去讨论这些东西呢？原因是什么呢？

P: 嗯，就是，不太了解这个概念吧。然后也没有就是发生类似的例子，嗯，很少发生这种需要讨论的场合。嗯，对，就是这样。

<**Participant #** 45. > - § 1 reference coded [1.70% Coverage]

Reference 1 - 1.70% Coverage

I: 那有没有和你的家庭正式的一起讨论过？

P: 没有。

I: 为什么呢？

P: 嗯，嗯，还是刚才我说的，没有这个意识吧。没有什么场合或需要要求我们必须正式讨论这个家族病。所以，嗯，只是闲聊吧。

<**Participant #** 46. > - § 1 reference coded [3.45% Coverage]

Reference 1 - 3.45% Coverage

如果让你提供精确的家族病史的信息的话，那您觉得什么是你的障碍？比如说像你刚刚提到的，你都没见过你的外婆。这是一种障碍，还有没有其他的？

P: 有可能大家从来没有提起过，没谈过这个，没有交流。

P: 还有一个是可能是你觉得也不太重要，你也不会问。但至于父母和兄弟姐妹就不一样了，你会觉得很重要，你会问。

I: 还有其他的么？

P: 还有就是你可能也没有这方面的知识认为去问这个很重要。大家没有想到我要，我要询问这个 ，想要了解这个。了解这个有什么重要意义。可能没有认识到这点
