# Supplementary material for: Chinese Americans’ Views and Use of Family Health History: A Qualitative Study
Source: PLoS One. 2016 Sep 20;11(9):e0162706. doi: 10.1371/journal.pone.0162706 (PMC5029932; doi:10.1371/journal.pone.0162706)
Supplement: S1 File — (ZIP) [file pone.0162706.s001.zip › Data/Barriers to discuss with family members/Physical separation with their family members.docx]

**Name:** Physical separation with their family members

**<Participant # 01. > - § 1 reference coded [2.51% Coverage]**

**Reference 1 - 2.51% Coverage**

P:我父亲我知道的较详细，我母亲去世得早，我才十一岁。我外祖父八十多岁去世，我也清楚。还有阿姨也是知道的。但父亲在大陆的那边完全不知道。我父亲我比较清楚，他去世时我五十岁。我母亲去世的早。我小时候的资料也保持的齐全。（**I:**为什么妈妈的那边不知道？他们在大陆？）P: 妈妈亲人都来了台湾。我阿姨健康怎样我也知道。（I: 那就是说，你没有收集家族病史的原因是因为你父亲的家人在大陆，母亲早逝。）

**<Participant # 08 > - § 2 references coded [2.07% Coverage]**

**Reference 1 - 1.75% Coverage**

I: 什么时候？

P: 在一起，都没话聊的时候，呵呵。

I: 所以说，你有没有办法说一个月一次，或是一年一次？

P: 哦，最多一年一两次。你看我家人大部分都在台湾哪，在讲电话的时候也不会讲到这些啊。

I: 那每次在聊的时候都很短吗。有没有五分钟？

P: 最多就是说一下，刚好是，舅妈有什么，有什么毛病。 帕金森disease, 哦，这样子哦，他们家有没有这个问题啊，就是这样子，都很短啊，没有特别去。

**Reference 2 - 0.32% Coverage**

I: 所以你认为没有办法跟你家人讨论家族病是因为他们在台湾。

P: 对，

**<Participant # 09. - § 1 reference coded [1.71% Coverage]**

**Reference 1 - 1.71% Coverage**

I: 那你有没有和你的家庭一起讨论你的家族病史么？有没有？

P: 都很少的。因为我们现在在这边，都有我们两个在美国么。

I: 是因为，原因是两个都在美国么？

P: 是。

**<Participant # 10. > - § 1 reference coded [3.46% Coverage]**

**Reference 1 - 3.46% Coverage**

I: 是因为，原因是两个都在美国么？

P: 是。

I: 所以才比较少讲这种事？

P: 儿子比较会讨论，和上面不讨论。

I: 两个小孩都是医生么？

P: 对。女儿是外科，儿子是，都分隔太远。所以和兄弟姐妹不讨论，和儿子会讨论。

I: 那么多久会讨论一次？

P: 没有事就没有事，有事就打电话过去。

I: 所以你那些都不是家族病史哦？

P: 不是家族病史。

**<Participant # 14 > - § 1 reference coded [1.40% Coverage]**

**Reference 1 - 1.40% Coverage**

（I：为什么不常常耳提面授？）P:以前常写信，现在都不写了。刚来美国是每月都写一封信回去，现在淡下来了，每年一通电话就算了。打电话就不能详细说，提一下吧。

**<Participant # 15. > - § 1 reference coded [2.28% Coverage]**

**Reference 1 - 2.28% Coverage**

P:他们全家都在香港，只有她自己在中国，她是青年团。）（笑）我从初中就离开家里。（I：因为你不和家人住在一起，你没收集家族病史？）P:也没有必要收集。在中国没有必要，没有可能。那时候在中国他们邮寄一本书给我也被没收。完全封锁了消息

**<Participant # 18. > - § 1 reference coded [2.63% Coverage]**

**Reference 1 - 2.63% Coverage**

P: 很久，大概一两年。通常我们都很少看医生，因为都没有什么病，所以也不会搬出来讨论。而且我跟父母也没住在一起。然后没什么大病啊，也不会在打电话的时候搬出来讲吧。报喜不报忧嘛。通话的时候如果是小事情通常不会搬出来讲。

I: 也就是没讨论的原因主要是因为大家距离很远。

P: 没住在一起。

**<Participant # 21. > - § 1 reference coded [1.20% Coverage]**

**Reference 1 - 1.20% Coverage**

I: 那你认为你没有经常讨论的障碍是什么呢？

P: 碰不到就不讨论了。你想我姐姐在上海我在这里。本来就是很少打电话。所以打起电话就会相互说一下。

**<Participant # 25. > - § 2 references coded [1.64% Coverage]**

**References 1-2 - 1.64% Coverage**

I: 那你有没有很刻意地去搜集家族病史的信息呢？

P: 没有。

I: 为什么没有去收集呢？

P: 恩，就是，当时年纪太小了，现在出来了，很多东西都不方便。距离的太远了。

**<Participant # 26. > - § 1 reference coded [1.03% Coverage]**

**Reference 1 - 1.03% Coverage**

I: 就是说，其实他们看见你的时候都会说到这个。所以你们没有讨论这个遗传病的原因就是你们不是住在一起。你觉得是这样么？

P: 是。

**<Participant # 28. > - § 2 references coded [4.77% Coverage]**

**Reference 1 - 3.64% Coverage**

P：我的家人和我的那些叔叔阿姨都住在加拿大或者香港， 我能知道的只是从我的父母口中听过谁谁有过什么病，但没有很详细。只是提到过他们有过什么病。没有提到什么时候去世。像提到过我的姨妈， 说她有癌症， 病了多少年。但我不知道详细情形。

I: 您说没有收集过“家族病史”的相关信息， （P：你是说亲戚等的信息吗？I:包括家族中所有的人，）为什么您不收集？

P：没有这种需要。除了至亲的父母，兄弟姐妹，叔叔伯伯阿姨这些人就没有收集过。

**Reference 2 - 1.13% Coverage**

I:您认为和您的家庭讨论您的“家族病史”的障碍是什么?

P：祖父母不再世，而且父母和其他家人也在香港或加拿大。很少会在一起讨论。

**<Participant # 48. > - § 1 reference coded [6.29% Coverage]**

**Reference 1 - 6.29% Coverage**

I: 所以你有没有和你的家庭一起讨论你的家族病史？

P: 嗯。。。讨论过，像我弟弟，他的青光眼。青光眼的事情，我们就讨论过，看看到底怎么回事？考虑到可能我的外婆，因为我的外婆眼睛瞎了。眼睛瞎了说是白内障瞎的。白内障瞎的，是不是有青光眼我们不知道。但结果就是眼睛瞎了。而且，我妈的眼镜也不舒服，也是有点，轻微的青光眼，现在又到我弟弟这里了，所以，高度怀疑是家族史。但是我们谁都不知道。

I: 大概多久和你弟弟讨论？

P: 就是他不舒服的时候就跟我讨论。没有不舒服就不讨论。

I: 有没有那种频率？比如说，你可能一年讨论那样一次，两次。

P: 很少。他就是工作很辛苦的时候，才发作。然后聊到了就聊到了。他，他不辛苦的话，不发作，我们就不聊。

I: 所以，就是，大概可以讲一两年吧，一两年一次这样子。

P: 差不多吧。是不是遗传我们都不知道。所以没有人去做。

I: 都没有跟人讨论过是不是遗传。

P: 因为讨论一个遗传病，医学上来讲，是一个非常严肃的。 你要做谱系调查的，是不是真的遗传。可是你现在，在外婆那儿，根本就不知道是不是有青光眼。就算知道，你也不可能就跑过去看呢！因为隔的很远啊。所以，哦，可能你外婆怎么怎么样，你妈妈怎么怎么样。。。

I: 所以就觉得，就是，你没办法和家人讨论家族病史的障碍，就是太远，所以很多事情就不知道了。然后。。。

P: 这是一个迁徙的缘故。就是，这个，居住，住处的缘故。再个就是，医疗，医疗。。。怎么说，医疗条件的缘故。比如说，你听说，听说我可能有家族病，你如果不是非常，那个关注的话，你也不可能去把你妈妈叫过来，把你爸爸叫过来看一下，所以说，也只是说，也就是口传，并不是那种非常serious的，就是说。。。明白么？

I: Okay. Okay. 那，所以，你从来没有跟你的家庭医生讨论过你的家族病史。
