# Supplementary material for: Chinese Americans’ Views and Use of Family Health History: A Qualitative Study
Source: PLoS One. 2016 Sep 20;11(9):e0162706. doi: 10.1371/journal.pone.0162706 (PMC5029932; doi:10.1371/journal.pone.0162706)
Supplement: S1 File — (ZIP) [file pone.0162706.s001.zip › Data/Barriers to discuss with family members/Self-defined healthy family.docx]

**Name:** Self-defined “healthy family”

**<Participant # 02. > - § 2 references coded [2.10% Coverage]**

**References 1-2 - 2.10% Coverage**

I: 为什么你不会跟你的家族去讨论这个家族病史？

P: 因为没有这个必要去讨论。大家都很健康，小孩子也很健康。好像没有必要去知道我们的，我们家的家族史，因为整个家都很健康。

I: 所以你的意思是没有必要，因为很健康。

P: 对，好像是没有必要，因为不需要。如果有需要的话，我就会探讨，打听，但目前还没有这个需要。

I: OKAY。那你只是你妈妈那边有高血压，心脏病这样？

P: 对。

**<Participant # 03. > - § 2 references coded [0.44% Coverage]**

**References 1-2 - 0.44% Coverage**

I: 为什么，为什么你没有跟他们讨论家族病史？

P: 因为我没有生病啊。很健康，就不会很主动啦。

**<Participant # 04. > - § 1 reference coded [0.53% Coverage]**

**Reference 1 - 0.53% Coverage**

I: 那你觉得为什么没有经常谈论这个家族病史呢？

P: 因为真的没有。比较幸运。

**<Participant # 08 > - § 3 references coded [1.99% Coverage]**

**References 1-2 - 1.72% Coverage**

哦，最多一年一两次。你看我家人大部分都在台湾哪，在讲电话的时候也不会讲到这些啊。

I: 那每次在聊的时候都很短吗。有没有五分钟？

P: 最多就是说一下，刚好是，舅妈有什么，有什么毛病。 帕金森disease, 哦，这样子哦，他们家有没有这个问题啊，就是这样子，都很短啊，没有特别去。

I: 所以你认为没有办法跟你家人讨论家族病是因为他们在台湾。

P: 对，另外一方面，对我们来说不是很重要的事情。

**Reference 3 - 0.27% Coverage**

I: 还有么？

P: 我觉得我们是公开的，大概就这样吧。

**<Participant # 11. > - § 2 references coded [8.62% Coverage]**

**References 1-2 - 8.62% Coverage**

I: 您从哪里得到的有关您的“家族病史”的信息？

P： 很小的时候从父母口中得知。因为我小时候吃一点（容易上火）的东西喉咙就疼。我妈妈跟我说我们的家族都这样不能吃太多。（I： 有看过医生吗？还是家里人这样说而已？） （I：有没有医生证明你不能吃这些东西？）**P:**医生也是知道的。医生能看出来的。医生不能说这个活，这是犯罪的。医生只是治病。（I：是不是医生跟你说这是遗传病？）P:不是，医生不会这样说这是遗传病。就算知道这是遗传病也不会说的。这是医生的医德。（I：为什么医生道德就不会说？）**P:**医生不能跟病人说他的遗传病。医生只是治病的。好像你是教授就是教授，你放问我你就是访问我。他只是治疗某个人就是这个人，治疗某种病就是某种病。跟私人的事没有关。（I：这不算事私人的事。）**P:**医生就是只说这个病。他所负责的病。（I：医生有没有跟你说这是遗传病？）**P:**没有。（I：这是你自己觉得）是的。遗传病多半是从family tree 哪里知道的，不是医生说的。我相信80% 的医生都不会说的。

I:您和您的家庭一起讨论您的“家族病史”么？

p： 有的。（I：多久讨论一次？）不会很经常。当我家我姐姐家里有发生这个事时。就会想到这是遗传下来的。一年一次吧。

I: 您认为和您的家庭讨论您的“家族病史”的障碍是什么? 像打电话时不想说，他们很忙。

P：不说的，他们都知道了。不会常常说，这是小事。

**<Participant # 13. > - § 1 reference coded [2.63% Coverage]**

**Reference 1 - 2.63% Coverage**

I: 您和您的家庭一起讨论您的“家族病史”么？多久讨论一次？

P：没有。（I：从来没有谈过？）**P:** 没有。多数是我的奶奶，姑妈在谈论， 他们不跟我们说的。（I：为什么？）**P:**他们觉得我们比较健康，不用跟我们说，而且他们不想让我们知道我们（会有）这种病，因为我们都长大了。

**<Participant # 24 > - § 2 references coded [3.78% Coverage]**

**References 1-2 - 3.78% Coverage**

I: 为什么没有去收集呢？

P: 原因？因为我爷爷奶奶都还在，最亲的都还在，所以也不会太关注这些事情。

I: 你是说从来都没有去收集过？

P: 嗯，应该是没有去搜集过。因为我的家人都在。

I: 你是从哪里知道这个家族病的信息的，比如说你妈妈头痛啊。

P: 因为我妈妈说她经常头痛。

I: 其实你妈妈没有给你说这是遗传病，只是你觉得。

P: 没有。

**<Participant # 29 > - § 2 references coded [5.32% Coverage]**

**References 1-2 - 5.32% Coverage**

I:为什么不呢？你的家族很明显有高血压遗传。

Ｐ：我觉得没有必要，如果是心脏病，癌症，可能要坐下来讨论一下。但高血压这是很平常的事（病）。大家都知道医学上是可预防。而且高血压现在通过吃药可以控制，不需要坐下来讨论。象我爸爸，他没有吃（降压）药，我问他怎样控制他的血压，他说他吃田七，啊，不，是灵芝。他说吃灵芝。灵芝不是专门降压药。可能他血压不是很高，所以灵芝就可以控制。我妈（病）比较厉害，所以她要吃西药，专门控制血压的药。（I：不严重，也就没必要讨论？）**P:**讨论也没用。还有，（语气转神秘）我相信有些事你做不做没有区别，是命运。（I：是注定的？）**P:**是注定。这不是科学可解释的。说起来，我的家族病史（高血压）算是很好了。

**<Participant # 33. > - § 1 reference coded [4.92% Coverage]**

**Reference 1 - 4.92% Coverage**

I：既然重要，您自己有没有收集过“家族病史”呢？有没有很认真地list出来过呢？

P：那倒没有。打听过，关心过而已。因为祖父母去世很早，我也不是很清楚他（她们）去世的原因，奶奶可能是得哮喘或和肺有关的病走的。姥爷可能是得癌症去世的，因为自己平时特别注重健康，所以没有很紧张自己会得什么和遗传有关的病。另外我家里有七个舅舅，五个近视，我觉得这应该和遗传有关，但在我们这代人里倒没反应出来。

**<Participant # 36 > - § 2 references coded [2.05% Coverage]**

**References 1-2 - 2.05% Coverage**

I: 如果是您搜集过家族病史相关的的信息，为什么您会这么做？刚才您也回答过，您好像没有怎么收集过，为什么会这样呢？

P: 没有，这本身没有听说过这样子的一个状况。

I: 啊，没有特别严重的疾病，疾病史，就好像不是非常的严重，必要，是这样子么？

P: 对。

**<Participant # 43 > - § 1 reference coded [3.35% Coverage]**

**Reference 1 - 3.35% Coverage**

P: 嗯。第一点，因为我们年纪还不到么。那第二点是，台湾的情况还不太一样。因为这些都是已经发生的，都是在台湾，而且我已经来这儿这么就了，和那边已经脱节了。那第三点就是，当时在台湾的时候，因为不是大房子，所以大家都分开住。分开住就会把亲戚这种亲密的关系疏远一点。这也有影响。所以，如果父母不提的话，我们也不会知道。那有时候，父母也觉得没有必要提，因为父母也觉得没有必要把病痛的东西让小孩子知道。这也是习惯啊，我们中国人的风俗习惯。

**<Participant # 44. > - § 2 references coded [7.22% Coverage]**

**Reference 1 - 5.64% Coverage**

I: 那你觉得让你提供精确的家族病史的信息的话，那您觉得什么是你的障碍？

P: 障碍，嗯，就是，从小，没有，从小很少得到这方面的信息吧。

I: 嗯。

P: 就是父母也不大给我讲这些，而且医生，平常看比较regular的病，也没有。也可能和我家庭有关系吧。

I: 嗯。

P: 主要是没有这方面的信息吧。

I: 那也就是你从来没有收集过这方面的信息，对吧？

P: 我没有收集过。

I: 那没有收集过的原因是什么呢？就是障碍是什么呢？

P: 嗯。。。

I: 像刚才说到的，来源于父母和医生的这方面的信息就比较少，那还有没有其他的？

P: 嗯，感觉上没有。

**Reference 2 - 1.59% Coverage**

I: 那是为什么没去讨论这些东西呢？原因是什么呢？

P: 嗯，就是，不太了解这个概念吧。然后也没有就是发生类似的例子，嗯，很少发生这种需要讨论的场合。嗯，对，就是这样。
