# Supplementary material for: Chinese Americans’ Views and Use of Family Health History: A Qualitative Study
Source: PLoS One. 2016 Sep 20;11(9):e0162706. doi: 10.1371/journal.pone.0162706 (PMC5029932; doi:10.1371/journal.pone.0162706)
Supplement: S1 File — (ZIP) [file pone.0162706.s001.zip › Data/How important to collect FHH/Important_anonymous.docx]

**Name:** Important

**<Participant # 01. > - § 1 reference coded [0.70% Coverage]**

**Reference 1 - 0.70% Coverage**

I: 您认为收集“家族病史”的相关信息重要吗？

P: 在诊断上是重要的。因为这些资料在正确诊断病情是重要的。

**<Participant # 02. > - § 1 reference coded [1.48% Coverage]**

**Reference 1 - 1.48% Coverage**

I: 那你认为收集相关家族病史的信息重要么？

P: 收集？嗯，如果，身为一个家族的member的话，是要知道那个家族的history。我觉得应该知道。可以prevent，不要等到发生了以后，才知道有这个家族史的话，有一点太晚。

I: 所以你觉得还是重要。

P: 对。

**<Participant # 03. > - § 3 references coded [1.26% Coverage]**

**Reference 1 - 0.26% Coverage**

I: 那你认为搜集家族病史重要么？

P: 我觉得很重要。

**Reference 2 - 0.24% Coverage**

I: 为什么？

P: 因为自己年纪大了，会开始担心。

**Reference 3 - 0.76% Coverage**

I: 你的意思是如果知道家族病是的话，你就可以去准备一下去预防，对不对。

P: 对，比如就是哪一年去看医生，去跟医生讲，然后每年的检查，我会去做抽血，去做特别的检查。

**<Participant # 04. > - § 1 reference coded [0.50% Coverage]**

**Reference 1 - 0.50% Coverage**

I:那您认为这个收集家族病史的这个资料，重不重要呢？

P: 是啊，我觉得是的。

**<Participant # 05. > - § 1 reference coded [0.34% Coverage]**

**Reference 1 - 0.34% Coverage**

I:那你认为收集家族病史的信息中重要么？

P: 重要。

**<Participant # 06. > - § 2 references coded [1.90% Coverage]**

**Reference 1 - 0.30% Coverage**

I: 那你认为收集家族病史的信息重要么？

P：当然重要哦。

**Reference 2 - 1.60% Coverage**

I: 你有没有收集过你的家族病史？

P:我就从陪他去开刀之后，我就非常小心。我就想啊，我妈妈他们的兄弟姐妹全部是糖尿病。包括我妈妈是胰脏癌，这个也是糖尿病来的。

I: 所以你有搜集过，这样？

P：我自己有积累这些资讯。其实我对医疗资讯非常非常感兴趣。个人的兴趣的问题啦。然后我爸爸，我也去研究。像我身上，这叫什么，这叫做遗传性多发性神经瘤。

**<Participant # 08. > - § 1 reference coded [0.26% Coverage]**

**Reference 1 - 0.26% Coverage**

I: 那你认为搜集这个重要么？

P: 嗯。。。应该也是吧，

**<Participant # 09. > - § 2 references coded [3.08% Coverage]**

**References 1-2 - 3.08% Coverage**

I: 那你认为有没有很重要去收集？

P: 我觉得还是很重要。因为家族病史它还是有遗传性或者延续性，那你这个整体看的话，你就知道哪些人有能有这种病，你就可以来预防它。我是从大的观点来看。所以说我觉得是重要。我爸爸这样，所以我就想提早去检查。要不然我这一辈子都不可能去检查。可是我就是不想去做。因为家里如果有人生了病，也想去做看一看。我姐姐她有跟我讲要去做。

**<Participant # 11. > - § 1 reference coded [0.45% Coverage]**

**Reference 1 - 0.45% Coverage**

I: 您认为收集“家族病史”的相关信息重要吗？

P：重要。

**<Participant # 13. > - § 1 reference coded [1.47% Coverage]**

**Reference 1 - 1.47% Coverage**

I: 您认为收集“家族病史”的相关信息重要吗？

P: 紧要，好像我的哥哥，当他长大，他会有自己的家庭。你要知道这种信息，所以你不会遗传给你的儿子。

**<Participant # 15. > - § 1 reference coded [0.67% Coverage]**

**Reference 1 - 0.67% Coverage**

I:您认为收集“家族病史”的相关信息重要吗？

P:重要。

**<Participant # 16. > - § 1 reference coded [3.86% Coverage]**

**Reference 1 - 3.86% Coverage**

I:您认为收集“家族病史”的相关信息重要吗？

P：我没有收集过。（I：你觉得重要吗？）有病，有事就重要，没事就不重要。（I：不是说你有什么病，而是说你的爷爷，或爸爸妈妈，或叔叔有这种病，你觉得收集这方面的信息重要？）重要的。 因为如果有这种病，那是要命的。

**<Participant # 17. > - § 2 references coded [1.35% Coverage]**

**References 1-2 - 1.35% Coverage**

I: 您认为收集“家族病史”的相关信息重要吗？

P:很重要。但我没有时间去做这个事。（I：你没有收集过。）没有。

**<Participant # 19. > - § 1 reference coded [0.90% Coverage]**

**Reference 1 - 0.90% Coverage**

I: 那您认为这个收集家族病史的这个资料，重不重要呢？

P: 当然重要。

**<Participant # 20. > - § 1 reference coded [1.05% Coverage]**

**Reference 1 - 1.05% Coverage**

I: 您认为搜集家族病史的信息重要么？

P: yeah。这个病啊，越早越容易。我这个病，如果早一点看医生。我二哥早就出来了，就是不懂这个东西。我二哥来到美国，我看到他也有啊。

**<Participant # 21. > - § 1 reference coded [1.17% Coverage]**

**Reference 1 - 1.17% Coverage**

I: 那您认为这个收集家族病史的这个资料，重不重要呢？

P: 应该是比较重要的。那就是，如果你知道家族中有什么病的话，你就可以提前去注意。

**<Participant # 22. > - § 1 reference coded [3.04% Coverage]**

**Reference 1 - 3.04% Coverage**

I: Do you think if it is important to collect your family health history information? If so, have you collected your family health history information?

P: yes, I think it’s pretty important to collect it. (I: so have you ever collected any information?) Yes, I have.

**<Participant # 23 > - § 1 reference coded [1.05% Coverage]**

**Reference 1 - 1.05% Coverage**

I: 那您认为这个收集家族病史的这个资料，重不重要呢？

P:重要。对医学研究有帮助。

**<Participant # 24 > - § 1 reference coded [1.10% Coverage]**

**Reference 1 - 1.10% Coverage**

I: 那您认为这个收集家族病史的这个资料，重不重要呢？

P: 搜集家族病史的信息？应该是重要的，非常重要。

**<Participant # 25. > - § 2 references coded [1.61% Coverage]**

**References 1-2 - 1.61% Coverage**

I: 那您认为这个收集家族病史的这个资料，重不重要呢？

P: 重要。

I: 你为什么觉得重要呢？

P: 对病能够有所了解吧。对自己，孩子啊，对父母，有所帮助吧。

**<Participant # 26. > - § 1 reference coded [1.85% Coverage]**

**Reference 1 - 1.85% Coverage**

I: 那您认为这个收集家族病史的这个资料，重不重要呢？就是说你知道你的家族里面有谁得过什么样的病，话说你的爷爷呀，你爷爷的兄弟姐妹呀，你的ante, uncle呀，有什么样的疾病，你认为这样收集一下重要吗？

P: 我想是重要，不过有时候很难去知道。

**<Participant # 27 > - § 2 references coded [1.76% Coverage]**

**Reference 1 - 1.35% Coverage**

P： 我知道有的人从小就得糖尿病，但是他到五六十岁，七八十岁，他还可以控制住。但有的人不注意控制，知识很少，他得了病也还不知道。或者高血压，从来不去看医生。

I: 那您认为这个收集家族病史的这个资料，重不重要呢？

**Reference 2 - 0.42% Coverage**

P: 确实重要。但是，恩。。。

I: 你有没有收集。

**<Participant # 28. > - § 1 reference coded [0.95% Coverage]**

**Reference 1 - 0.95% Coverage**

I: 您认为收集“家族病史”的相关信息， 譬如你的家族中人有什么相同疾病的信息，重不重要？

P：挺重要的。

**<Participant # 29. > - § 2 references coded [1.57% Coverage]**

**References 1-2 - 1.57% Coverage**

I: 您认为收集“家族病史”的相关信息重要吗？

Ｐ：重要，绝对重要。（I：为什么？）这是一个根据，你有家族遗传病，也就是你有可能遗传到，医生会根据你的家族病史来判断你的病情。

**<Participant # 30. > - § 1 reference coded [0.77% Coverage]**

**Reference 1 - 0.77% Coverage**

I: 您认为收集“家族病史”的相关信息重要吗？

P: 当然重要。

**<Participant # 31. > - § 1 reference coded [1.88% Coverage]**

**Reference 1 - 1.88% Coverage**

I：好，下一个问题问你认为收集“家族病史”的information重要吗？

I：那你觉得很重要，你有没有收集过你的“家族病史”的信息？

P：一般都是informal 的，我倒是和妈妈打听过我姥姥的病是怎么回事，我自己非常有concern, 但是我没有具体到像您今天这样问过。

P：我觉得挺重要的。

**<Participant # 32. > - § 1 reference coded [1.11% Coverage]**

**Reference 1 - 1.11% Coverage**

I：您认为收集“家族病史”的相关信息重要吗？如果重要，您有没有收集过您的“家族病病史”的信息？

P: 我没有特殊收集过，但我觉得很重要。不然我也不会答应你这个appointment.应该对自己的健康有个最基本的了解。

**<Participant # 33. > - § 2 references coded [2.68% Coverage]**

**References 1-2 - 2.68% Coverage**

I：您认为收集“家族病史”的相关信息重要不？如果重要，您有没有收集过您的“家族病史”信息？

P:这个我认为是非常重要。因为我本人是学医的，几乎所有的病人看病时都会被问到家里有没有什么遗传病啊，有没有什么家族病。

**<Participant # 34. > - § 1 reference coded [13.21% Coverage]**

**Reference 1 - 13.21% Coverage**

I：我想问您收集家族病史的信息重要吗？我们做这份访谈主要是希望从在美的亚裔身上得到第一手的资料，希望亚裔作为少数族裔的健康状况可以得到主流社会更多的关注。

P：我觉得这种收集是非常重要的。对于华人来讲，一般是分为两种情况，一部分是有非常好的教育，生活环境良好；他（她）们的健康状况相对好些，而且他们的医疗保险和收入状况较好；但是另外一部分华人呢，就是我们说的打工族，他（她）们来美国之后呢，由于本身的教育背景，或者语言能力等因素，他们所经过的体力劳动的过程，也就是说他们在追寻美国梦的过程中，对于自身疾病和健康状况的了解和常识都不如那受到良好教育的人。有些人根本没有医疗保险，因为收入低，英文不好，找不到合适的医生治病。在我自己行医的过程中，我见到过这类的人群，这种困难重重的患者人数不少。所以觉得这种信息采集是非常重要的。我们华人作为少数裔的健康状况如果能够被关注，而且能够有渠道对他（她）提供帮助是很重要的。有一个公司的代表，礼拜三下午和我刚刚讨论完，要做普及华裔社区对乙型肝炎的教育，他说中国人乙型肝炎的感染率非常高，但很多人从来没有诊断过，根本不知道自己得了肝炎，最后到了肝衰竭的时候已经晚了。我有一个患者是餐厅的服务员，他最后是肝腹水才到我这儿来，我已经很难有办法去帮他了，另外他又没有保险，所以费用由没人能掏。所以我觉得你这个做得非常非常重要。还有一个误区，华人社区的人小病不治，大病又治不了的时候是在是太多了。我觉得我们华人一直是弱势群体，语言上不去，文化也上不去，我觉得应该呼吁更过的社会力量关注我们的健康。

**<Participant # 35. > - § 1 reference coded [1.27% Coverage]**

**Reference 1 - 1.27% Coverage**

I: 那你认为收集家族病史的相关信息是不是很重要？

P：我认为很重要。因为你要是知道了你家族的病史的话，得到遗传的话，你会去注意它，关注它。尽量避免去得这个病。

I: 会有这个意识

**<Participant # 37. > - § 1 reference coded [2.53% Coverage]**

**Reference 1 - 2.53% Coverage**

I: 下个问题问一下，您觉得搜集家族病史的相关信息是否重要？如果重要的话，您个人有没有搜集过您的家族病史的相关的信息呢？

P：我觉得这个应该是越来越重要。以前我们觉得不是那么重要，那么现在的社会压力大了，疾病的发生的几率也高，应该是要考虑到这些方面，这些方面的信息，然后呢，早些做一些准备，防患于未然。应该是这样子。因为我们以前都不是太重视。

**<Participant # 38. > - § 1 reference coded [1.83% Coverage]**

**Reference 1 - 1.83% Coverage**

I: 好，那问一下，您觉得搜集家族病史的相关信息重要吗？

P：哦，那当然非常重要。

I: 那如果重要的话，您有没有搜集过您自己的家族病史的信息呢？

P: 对于我的家族病史的话，我可能会有一些了解。但是说，收集到非常精确的，还没有达到这样子的程度。

**<Participant # 39. > - § 2 references coded [1.50% Coverage]**

**References 1-2 - 1.50% Coverage**

I: 那您认为收集这个家族病史的信息重不重要？

**P:** 很重要。

I: 很重要。那您个人有没有搜集过这方面的信息？

**P**: 嗯，嗯，我没特意地搜集过。但是我大概知道。

I: okay

**<Participant # 40 > - § 1 reference coded [0.60% Coverage]**

**Reference 1 - 0.60% Coverage**

I: okay。那您认为搜集这种信息重不重要？

P: 重要。

**<Participant # 43 > - § 1 reference coded [1.51% Coverage]**

**Reference 1 - 1.51% Coverage**

I: 那您个人认为收集这个家族病史的信息重不重要，就是对人来讲？

P: Okay， 我看到了。我没有搜集过，我只是知道的，接触过的。

I: 那您觉得重要么？

P: 大概重要吧。

**<Participant # 44 > - § 1 reference coded [1.19% Coverage]**

**Reference 1 - 1.19% Coverage**

I: 下面是关于家族病史信息的。那您个人认为这个收集家族病史重不重要么？

P: 嗯，我，我认为很重要，但是接触的很少。

**<Participant # 45.> - § 1 reference coded [3.04% Coverage]**

**Reference 1 - 3.04% Coverage**

I: 好，那您个人认为收集家族病史重不重要？

P: 嗯，我认为比较重要吧。

I: 那为什么觉得它重要？

P: 嗯，嗯，就个人而言，可以对自己的家族病有所了解，嗯，能够做到心中有数吧。对于社会而言，对医学上来说，可以有利于家族病史的研究吧。

I: 嗯。

P: 嗯，能够让研究人员找到一些规律，能让，比如说得出一些结论，更好地用于人类疾病的预防和治疗。

**<Participant # 46 > - § 1 reference coded [3.24% Coverage]**

**Reference 1 - 3.24% Coverage**

I: 那您个人认为这个收集家族病史，这个课题重要么？

P: 当然重要。我认为比较重要。

I: 那为什么呢？

P: 你起码，起码，当然对个人来讲，不是对研究来说，对个人来讲，容易得哪些疾病，你自己很清楚。起码，就是说，你有个预防的措施，对吧？！

I: 嗯。那你觉得从research的角度有什么好处呢？

P: 那也是有好处啦。你可以知道一些pattern，你知道pattern的话，作为一个research来讲，对预防也是一种帮助吧。

**<Participant # 47. > - § 1 reference coded [3.86% Coverage]**

**Reference 1 - 3.86% Coverage**

I: okay.嗯。。。那您认为收集这个家族病史的信息重不重要？

P: 你是说我本人的？

I: 不是你本人，就是generally 认为搜集信息重要么？比如说你家人的，你的朋友的？

P: 朋友那种，我觉得也没有必要了解太多吧。毕竟比较privacy。

I: 那对于个人的呢？

P: 倒是我本人的话，嗯。。。就是我觉得了解一些，大概，就是说，自己会比较注意一点。你去医生那儿检查什么的，嗯，就是pay attention 吧，对一些相关的疾病会注意。

**<Participant # 48 > - § 1 reference coded [0.38% Coverage]**

**Reference 1 - 0.38% Coverage**

I: 那你认为搜集家族病来讲重不重要？

P: 嗯。。。当然重要。对医学工作者来讲当然是重要的

**<Participant # 49 > - § 1 reference coded [1.84% Coverage]**

**Reference 1 - 1.84% Coverage**

I: 那你认为搜集家族病来讲重不重要？

P: 挺重要的。我是从做研究的这个角度考虑的。我的一个做研究的朋友告诉我，掌握了一个家族病史，就像找到了一个宝藏。而且如果一个家都得肝癌，有的家就不让人知道，有的就像贡献出来，让人研究，想治愈这一方面。因为他们家族传下去还会有。
