# Supplementary material for: Chinese Americans’ Views and Use of Family Health History: A Qualitative Study
Source: PLoS One. 2016 Sep 20;11(9):e0162706. doi: 10.1371/journal.pone.0162706 (PMC5029932; doi:10.1371/journal.pone.0162706)
Supplement: S1 File — (ZIP) [file pone.0162706.s001.zip › Data/How important to collect FHH/Not important_anonymous.docx]

**Name:** Not important

**<Participant # 07.> - § 2 references coded [0.99% Coverage]**

**References 1-2 - 0.99% Coverage**

I: 那你认为搜集家族病史重要么？

P: 嗯。。。讲起来似乎重要啦。但是没有，但是知道之后，也不知道专门，可以干些什么事情，或者说是对我有什么帮助。

**<Participant # 10> - § 2 references coded [2.22% Coverage]**

**Reference 1 - 0.53% Coverage**

I: 那你认为有没有很重要去收集？

P: 没有。

**Reference 2 - 1.69% Coverage**

I：你认为不重要，那为什么认为不重要？

P：我觉得管太多了，反而心情会不好。我妹妹整天说，谁有怎么样，怎麽样，她就担心，我以后死掉的几率比别人大。我说没有这种事情，我不觉得别人跟你有什么关系。所以说我就说。

**<Participant # 14> - § 2 references coded [1.86% Coverage]**

**References 1-2 - 1.86% Coverage**

I: 您认为收集“家族病史”的相关信息重要吗？

P：我从来都没有这种认识。没有说有什么病就该做些什么。我从来没有这个认识。（I：因为没有认识，所以没有收集过。）没有。（笑）没有认识，怎会收集？

**<Participant # 18> - § 1 reference coded [1.61% Coverage]**

**Reference 1 - 1.61% Coverage**

I: 那你觉得收集这些信息对你来说重不重要？你需不需要记得我爸爸，爷爷，奶奶得过什么病，这些重要吗？

P: 曾经不重要。今天和你坐下来谈了之后，觉得还是很重要的。就是要留意以下了

**<Participant # 36. > - § 1 reference coded [1.41% Coverage]**

**Reference 1 - 1.41% Coverage**

I: 我想问一下您啊，从您个人的角度，您认为搜集家族病史的相关信息是否重要？如果重要的话，您个人有没有搜集过相关的信息呢？

P：嗯，我没想过这个问题，所以说我大概没有说很重要吧。

**<Participant # 42 > - § 1 reference coded [1.37% Coverage]**

**Reference 1 - 1.37% Coverage**

I: okay。那您认为搜集这种信息重不重要？

P: 嗯，我觉得不太重要。不一定完全，祖先的病也不一定完全地pass。
