# Supplementary material for: Chinese Americans’ Views and Use of Family Health History: A Qualitative Study
Source: PLoS One. 2016 Sep 20;11(9):e0162706. doi: 10.1371/journal.pone.0162706 (PMC5029932; doi:10.1371/journal.pone.0162706)
Supplement: S1 File — (ZIP) [file pone.0162706.s001.zip › Data/How important to collect FHH/Not sure_anonymous.docx]

**Name:** Not sure

<Participant # 12> - § 1 reference coded [1.12% Coverage]

Reference 1 - 1.12% Coverage

I: 您认为收集“家族病史”的相关信息重要吗？

Ｐ：这个我不太清楚。我不是医生。

<Participant # 41 > - § 1 reference coded [0.80% Coverage]

Reference 1 - 0.80% Coverage

I: okay。那您觉得搜集这个信息重不重要？

P: 搜集这些信息重不重要？？嗯，没有想过也
